# Magnetically steerable bacterial microrobots moving in 3D biological matrices for stimuli-responsive cargo delivery

**DOI:** 10.1126/sciadv.abo6163

**Published:** 2022-07-15

**Authors:** Mukrime Birgul Akolpoglu, Yunus Alapan, Nihal Olcay Dogan, Saadet Fatma Baltaci, Oncay Yasa, Gulsen Aybar Tural, Metin Sitti

**Affiliations:** ^1^ Physical Intelligence Department, Max Planck Institute for Intelligent Systems, 70569 Stuttgart, Germany.; ^2^ Institute for Biomedical Engineering, ETH-Zürich, Zürich 8092, Switzerland.; ^3^ Stuttgart Center for Simulation Science (SC SimTech), University of Stuttgart, 70569 Stuttgart, Germany.; ^4^ Department of Pharmaceutical Technology, Faculty of Pharmacy, Ege University, 35040 Izmir, Turkey.; ^5^ School of Medicine and College of Engineering, Koç University, 34450 Istanbul, Turkey.

## Abstract

Bacterial biohybrids, composed of self-propelling bacteria carrying micro/nanoscale materials, can deliver their payload to specific regions under magnetic control, enabling additional frontiers in minimally invasive medicine. However, current bacterial biohybrid designs lack high-throughput and facile construction with favorable cargoes, thus underperforming in terms of propulsion, payload efficiency, tissue penetration, and spatiotemporal operation. Here, we report magnetically controlled bacterial biohybrids for targeted localization and multistimuli-responsive drug release in three-dimensional (3D) biological matrices. Magnetic nanoparticles and nanoliposomes loaded with photothermal agents and chemotherapeutic molecules were integrated onto *Escherichia coli* with ~90% efficiency. Bacterial biohybrids, outperforming previously reported *E. coli*–based microrobots, retained their original motility and were able to navigate through biological matrices and colonize tumor spheroids under magnetic fields for on-demand release of the drug molecules by near-infrared stimulus. Our work thus provides a multifunctional microrobotic platform for guided locomotion in 3D biological networks and stimuli-responsive delivery of therapeutics for diverse medical applications.

## Teaser

Bacterial microrobots steered by magnetic fields navigate in 3D biological matrices for stimuli-responsive drug release.

## INTRODUCTION

Dexterous manipulation of tiny objects at the micro- and nanoscale to establish further understanding of active cargo delivery has been the cornerstone of microrobotic applications for minimally invasive therapies (*1*, *2*). Various microrobotic platforms have been developed, including fully synthetic micromotors actuated by external stimuli, such as magnetism (*3*–*5*), light (*6*), and acoustics (*7*, *8*). In contrast, biohybrid microrobots, which combine a motile microorganism (e.g., bacteria or algae) with an artificial component (e.g., micro/nanocarriers), are self-powered micromachines with intrinsic propulsion, sensing, and targeting mechanisms (*9*–*12*). Among different types of microrobots, bacteria-driven biohybrids stand out from the rest, considering their efficient flagellar propulsion, ability to navigate in hard-to-reach bodily tissues, and sensing capabilities of physiological and pathophysiological gradients (*12*, *13*). In addition, advances in genetic engineering tools for bacteria enable unprecedented capabilities to the biological units, including diminished cytotoxicity and expression of targeted surface molecules (*14*, *15*). Thus, bacterial biohybrids, particularly when decorated with multiple functional units, such as contrast agents, therapeutics, and targeting moieties, become ideal candidates for medical microrobot applications. However, most of the fabrication techniques used in the design of biohybrid microrobots have deleterious effects, which may interfere with the intrinsic properties of the microorganisms, including swimming speed, taxis, and membrane protein expressions, among others (*12*). In addition, smart and stimuli-responsive artificial units that can be used multimodally would boost the performance of a biohybrid microrobot by integrating additional functionalities, including spatiotemporal position control, enhanced tissue penetration, and microenvironment-induced or externally triggered cargo release. Thus, the selection of the artificial units (including their size, surface properties, payload efficiency, and stimuli responsiveness) and the conjugation route should be carefully evaluated to provide a multifunctional and high-performance biohybrid microrobot design.

A diverse selection of artificial units of various size, geometry, material, and function has been used in biohybrid microrobotic designs, including polymeric particles (*16*, *17*), polymer tubes (*18*), red blood cells (*10*, *19*, *20*), liposomes (*21*, *22*), and nanoparticles (*23*–*25*). The material, size, and shape of the artificial unit hold a key position in biohybrid microrobot fabrication, not only for the active propulsion and maneuverability but also for the optimum cargo-loading efficiency and penetration through biological tissues. Artificial units that are smaller than the bacterial cell dimensions (i.e., nanometer range) will induce less fluidic drag on the biohybrids, maintaining the natural swimming velocity (*19*, *21*) and allowing bacterial motion through the micro/nanopores of biological matrices. However, because smaller cargo carriers will contain less total volume for encapsulation, artificial units with higher loading efficiency coupled with a high-throughput conjugation scheme are prerequisites to engineer high-performance biohybrid microrobots. Nanoliposomes (NLs), which present high cargo loading efficiency along with tunable size, surface properties, and biocompatibility, have been recently used in bacterial biohybrid designs for the delivery of various molecules (*21*). Furthermore, by tuning their phospholipid membrane properties, NLs can be tailored to release their cargo upon exposure to local pH changes or external triggers, such as light (*26*). In addition, magnetic materials and nanoparticles (mNPs) are broadly integrated into biohybrid designs to achieve active magnetic steering using external fields (*10*, *11*, *16*, *23*, *27*). Magnetic field gradients and magnetic alignment can contribute to the navigation of the microrobots in tumor matrix models to increase the bioavailability of bacterial biohybrids at the target site and thus the drug molecules carried along. Despite the recent advances, multifunctional bacterial biohybrid designs with high payload efficiency, multimodal stimuli-responsive cargo release, and high fabrication throughput along with preserved bacterial motility and enhanced tissue penetration are yet to be achieved.

**Fig. 1.**
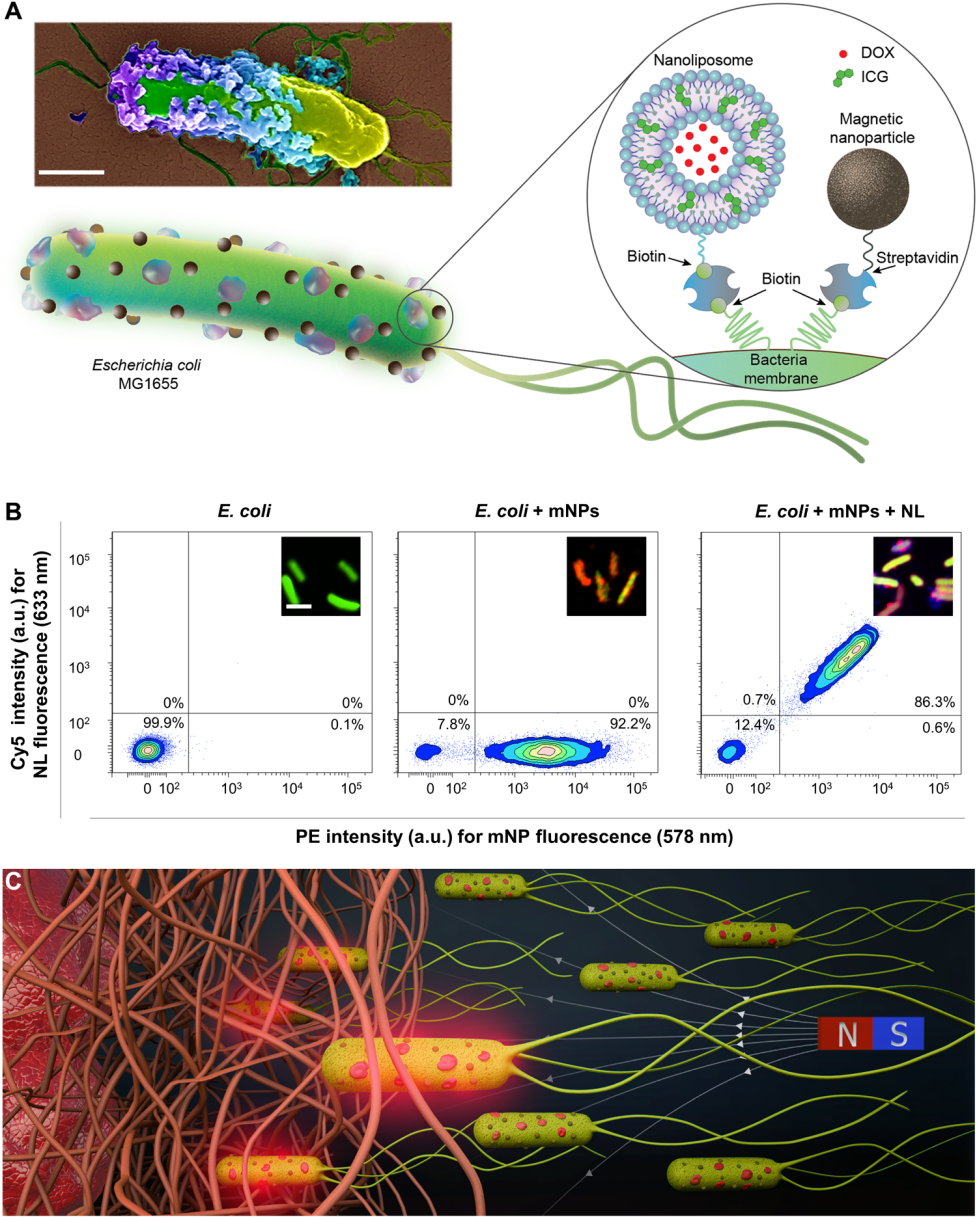
Bacterial biohybrids carrying mNPs and NLs. (**A**) Schematic illustration of the bacterial biohybrid microrobots, conjugated with NLs and mNPs. NLs are loaded with DOX and ICG, and both NLs and mNPs are conjugated to bacteria via biotin-streptavidin interactions. Inset shows an SEM image of an example bacterial biohybrid carrying mNPs and NLs. Image was pseudocolored. Scale bar, 500 nm. (**B**) Flow cytometry density plots of (i) free bacteria expressing GFP, (ii) bacterial biohybrids carrying mNPs tagged with red fluorescence, and (iii) bacterial biohybrids carrying mNPs tagged with red fluorescence and NLs tagged with Cy5, showing successful conjugations quantitatively. a.u., arbitrary units. Scale bar, 2 μm. (**C**) Conceptual schematics depicting bacterial biohybrid microrobots magnetically guided through porous microenvironments toward target tissues, such as a tumor. Bacterial biohybrids can release their payload upon NIR irradiation, enabling stimuli-responsive cargo release in 3D biological matrices. N, north; S, south.

Here, we report a biohybrid microrobotic platform composed of genetically engineered motile bacteria, mNPs for externally guided magnetic swimming control, and pH- and light-responsive NLs for on-demand cargo delivery in porous biological matrices. Bacterial biohybrids were constructed by using harmless, noncovalent interactions for the attachment of the synthetic materials, streptavidin mNPs, and biotinylated NLs to motile bacteria (Fig. 1A). Conjugations with high manufacturing yield and stimuli-responsive active cargo delivery were realized by engineering a liposomal formulation that encapsulates drug molecules [doxorubicin (DOX)] and photothermal agents [indocyanine green (ICG)]. Bacterial biohybrid microrobots maintained their original swimming velocity after the addition of these artificial units, averaging a swimming velocity of 18.5 μm/s, and their navigation was controlled and guided by external magnetic fields. Magnetically steered bacterial biohybrids were guided toward three-dimensional (3D) tumor spheroids in confined microchannels mimicking the vasculature networks, and magnetism-enhanced biohybrid motion in fibrous networks was demonstrated inside 3D collagen gels. Furthermore, on-demand release of anticancer drugs was realized by bacterial biohybrids localized on tumor spheroids through near-infrared (NIR) light activation. Overall, the bacterial biohybrid design presented here provides a systematic and high-throughput platform for multifunctional medical microrobots that can overcome biological barriers and perform stimuli-responsive active therapeutic release.

## RESULTS

### Preparation and characterization of bacterial biohbyrids

We chose a genetically engineered substrain of *Escherichia coli* MG1655 (*28*) as the biological unit, which expresses biotin attachment peptides and green fluorescent protein (GFP), enabling further functionalization of the cell surface sequentially with compounds of streptavidin and providing an effective means for fluorescence imaging (Fig. 1A). Optimizations regarding biotin attachment peptide and GFP expressions were conducted using different concentrations of isopropyl-β-d-thiogalactopyranoside (IPTG) and *L*-arabinose during bacterial cultivation, respectively, by checking the expression levels with fluorescence microscopy imaging and flow cytometry analysis during the growth phase of cultures (fig. S1). After optimizations, we used core-shell mNPs with covalently bound streptavidin to prepare magnetic bacterial biohybrids. Further functionalization with NLs was done via biotin-streptavidin-biotin binding complex, by simply incubating magnetic bacterial biohybrids in a streptavidin solution, followed by a second incubation in a biotinylated NL solution (Fig. 1A). Using biotin-streptavidin coupling allowed us to integrate artificial units onto viable bacteria without the need for harsh chemical or physical incubation processes that are required in most bacterial biohybrid conjugation approaches used in the literature (*29*–*31*). To characterize the efficiency of our coating method quantitatively, we performed flow cytometry analyses, which showed that 92.2% of the bacteria population (the number of biohybridized bacteria/total number of bacteria) was conjugated with mNPs (Fig. 1B). In addition, when the same analyses were performed on bacterial biohybrids carrying both mNPs and NLs, density plots indicated that 86.3% of the population was double positive for both artificial cargoes (Fig. 1B). Fluorescence microscope and scanning electron microscopy (SEM) images and swimming videos of bacterial biohybrids further corroborated the flow cytometry results, showing that a high portion of biohybrids had cell membrane coverage of both mNPs and NLs (Fig. 1B, fig. S2, and movie S1). Owing to the high coating efficiency, we hypothesize that bacterial biohybrids would be magnetically guided toward a target region with precision and release their cargo (i.e., drug molecules encapsulated within NLs) on-demand with external cues (Fig. 1C).

### Stimuli-responsive liposomal drug delivery platform

**Fig. 2.**
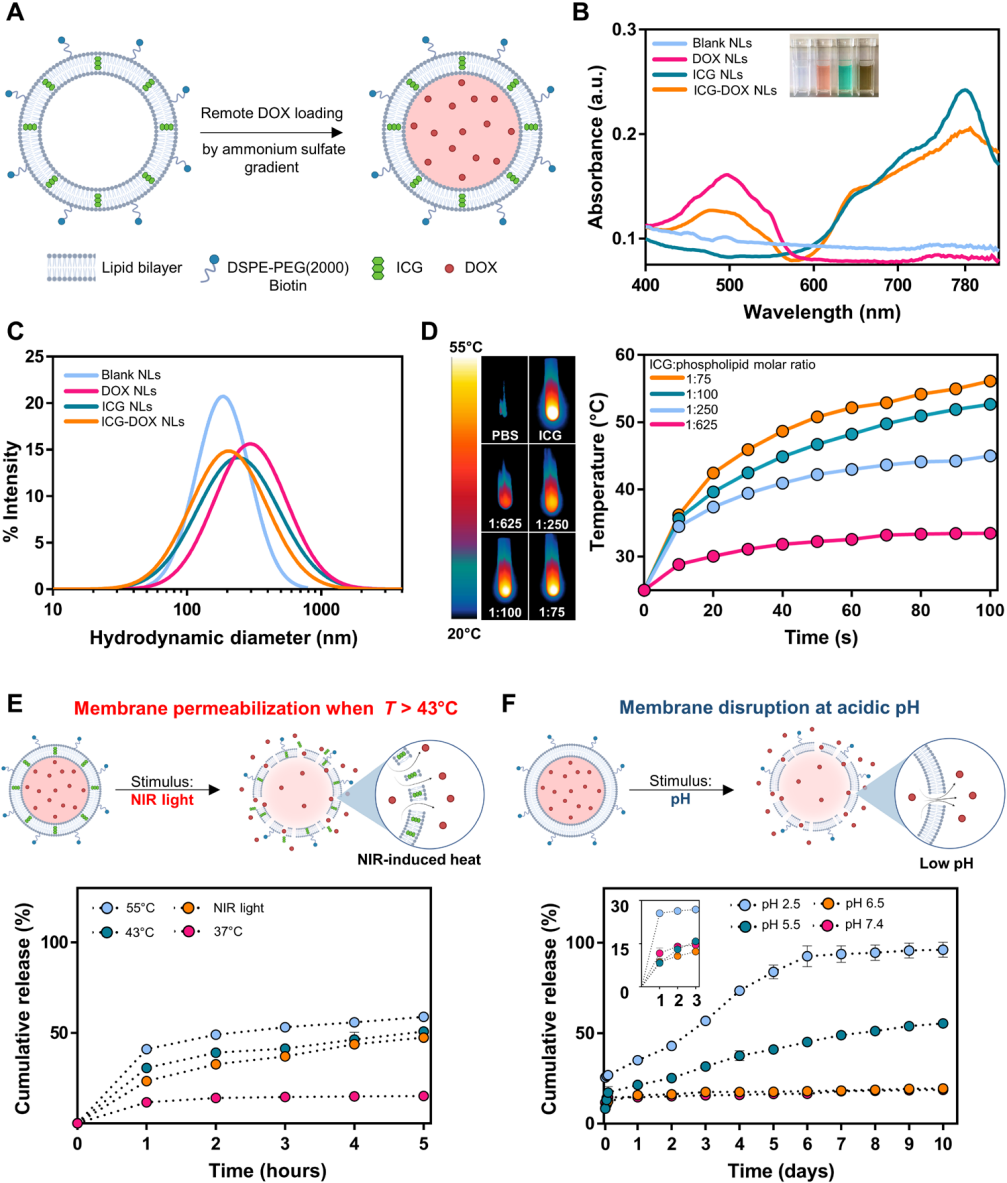
Synthesis and characterization of DOX- and ICG-loaded NLs. (**A**) Schematic of the NL synthesis. ICG is entrapped within the phospholipid bilayer of a NL, and DOX is remotely loaded via the ammonium sulfate gradient method to the inner aqueous core. (**B**) The absorption spectrum of blank NLs, DOX NLs, ICG NLs, and ICG-DOX NLs. The inset shows the NL solutions placed in PMMA cuvettes. (**C**) Size distribution of NLs measured by DLS. (**D**) Infrared thermal images and temperature profiles of various ICG NLs under NIR irradiation (~0.6 W/cm^2^, 100 s). Different ICG NLs were prepared by changing ICG:phospholipid molar ratio. (**E**) The NIR-driven cumulative drug release profile of ICG-DOX NLs over 5 hours at 37°, 43°, and 55°C and after NIR irradiation (~0.6 W/cm^2^, 5 min). At elevated temperatures (>43°C), lipid membranes start to disintegrate and become permeable, inducing DOX release. (**F**) The pH-driven cumulative drug release profile of DOX NLs over 10 days at pH 2.5, 5.5, 6.5, and 7.4. Low pH triggers membrane disruption and the release of DOX molecules. The inset shows cumulative drug release profile within the first 3 hours of the experiment.

Considering their versatility and multifunctionality, we selected a liposomal nanomedicine platform as the artificial cargo carrier unit of our bacterial biohybrid design. Specifically, we designed a photothermally active liposomal formulation with ICG embedded in the phospholipid bilayer that can absorb NIR light and convert it into heat, which ultimately triggers structural changes in the lipid membrane and the release of the intraliposomal content, i.e., chemotherapeutic DOX molecules (Fig. 2A). ICG, an amphiphilic medical fluorescent cyanine dye, was incorporated within the lipid bilayer of the NLs, whereas the hydrophilic form of DOX (doxorubicin hydrochloride) was encapsulated within the aqueous center of liposomes using the remote loading method driven by transmembrane ammonium sulfate gradient (Fig. 2A). With the remote loading method, a pH gradient is created with acidic conditions (pH 5.5) inside and neutral conditions (pH 7.4) outside of liposomes. When mixed with the blank liposome solution where the outer pH is 7.4, DOX is in the neutral form that can pass through the liposomal membrane and subsequently gets protonated at the intraliposomal space due to the low-pH conditions. DOX molecules are then bound with sulfate and form a complex where multiple DOX molecules bind to one another, making them more stable with minimal leakage to extraliposomal space (*26*). Drug loading optimizations were realized by changing the phospholipid concentration and drug to phospholipid ratio, and encapsulation efficiency (EE%) was calculated for each sample (table S1). Remote loading method enabled high encapsulation efficiency (86.1%) and sustained drug-liposome complex stability.

ICG and DOX loading was further characterized by absorption spectra measurements. All liposome solutions [blank NLs, only DOX loaded (DOX NLs), only ICG loaded (ICG NLs), and both ICG and DOX loaded (ICG-DOX NLs)] were loaded into clear measurement cuvettes, and absorbance values were recorded for 400- to 850-nm light wavelengths (Fig. 2B). The absorption spectra showed that DOX NLs had a strong absorption peak at 496 nm, which is in agreement with the absorption characteristics of free DOX. ICG NLs had an absorption peak at 780 nm, which also corresponds to the characteristic absorption peak of free ICG. Two strong absorption peaks were recorded at 782 and 488 nm for ICG-DOX NLs, which belong to the absorptions of ICG and DOX, respectively. Hydrodynamic diameters and zeta potentials of NLs were measured by the dynamic light scattering (DLS) method (Fig. 2C and table S2). While a slight increase in the hydrodynamic diameters was observed between blank NLs and ICG- and/or DOX-loaded NLs (from 190 to 210 nm to 205 to 289 nm), all liposomes had a positive zeta potential (7.6 to 8.4 mV; Fig. 2C).

To test the photothermal efficiency of ICG NLs, we continuously monitored the changes in temperature with an infrared thermal camera under constant NIR light irradiation. We prepared different liposomal formulations of ICG, simply by keeping the phospholipid concentration fixed and varying the ICG amount in each formulation, thus changing the ICG:phospholipid molar ratio (1:75, 1:100, 1:250, and 1:625), and irradiating the samples at a fixed power density of 0.6 W/cm^2^ for 100 s (Fig. 2D). Liposomal ICG formulations prepared with 1:75 and 1:100 ICG:phospholipid molar ratios were able to heat up to as high as 56.2°C from the room temperature (∆*T* = ~31°C) within less than 2 min (Fig. 2D), which is well above the cell-killing range that defines hyperthermia (*32*). In addition, ICG-DOX NLs were able to heat up to ~43°C from room temperature (∆*T* = ~18°C) within 3 min (fig. S3). The reason for the decreased end-point temperature reached by ICG-DOX NLs compared to ICG NLs (i.e., ~56° to ~43°C) could be attributed to the DOX loading process performed at an elevated temperature (60°C), where the phospholipid membrane becomes more fluid, allowing DOX uptake. However, already loaded ICG molecules are also released during DOX loading process at this elevated temperature, resulting in lower ICG concentration in the NLs and thus loss of photothermal conversion efficiency. Despite the decreased photothermal efficiency, the temperature increase of ~18°C would still facilitate efficient heating under physiological conditions for hyperthermia and heat-triggered release applications. To further test the feasibility of the photothermal heating scheme described here, we fabricated a physiologically relevant phantom tissue mimicking the light scattering and absorption characteristics of the dermis layer of the skin based on the previous literature (*33*, *34*). When ICG-DOX NLs were irradiated through the phantom tissue at the same power density (0.6 W/cm^2^), the temperature was elevated to the same levels (~43°C, ∆*T* = ~18°C; fig. S4) as in experiments without the phantom tissue (~43°C, ∆*T* = ~18°C; Fig. 2D), although taking ~7.5 min to reach the peak compared to ~3 min for experiments without the phantom tissue.

Two modes of drug release were investigated with the liposomal carriers. The first mode of the drug release, triggered by the NIR light irradiation, is based on the conformational changes that start occurring gradually before reaching *T*_m_, where the lipid bilayer transitions from the ordered gel phase to the disordered liquid-crystalline phase. NIR triggering showed three times higher release of DOX compared to the physiological conditions at 37°C and at pH 7.4 (Fig. 2E). Within the 5 hours after NIR light irradiation, around 50% of DOX was released, whereas only 15% of the drug was released from samples kept at 37°C without NIR light irradiation. To corroborate our results with the release trends at elevated temperatures, we also investigated drug release from samples kept at 43° and 55°C, which showed 51 and 58% drug release at 5 hours, respectively. Although we observed the highest release at *T*_m_ (i.e., 55°C), an increased drug release was also observed when the temperature was increased from 37° to 43°C due to the gradual increase in membrane fluidity of the NLs around 43°C.

In the second drug release mode, which is triggered by low pH, we analyzed the release of DOX molecules from NLs over 10 days at various pH conditions (ranging from 2.5 to 7.4) to evaluate the pH-dependent release mechanism (Fig. 2F). At pH 2.5, 98% of the drug was released within 6 days, with a burst release of approximately 30% within the first 3 hours. Drug release reached up to around 56% at pH 5.5 over 10 days, which showed a sustained release profile at a relatively low-pH condition. Drug release at pH 6.5 and pH 7.4 was relatively lower, with 19 and 17% cumulative DOX release over 10 days, respectively. This trend indicates a pH-dependent release mechanism, where a higher release of molecules is observed at lower pH. The multifunctional stimuli responsiveness of the artificial cargoes presented here renders the bacterial biohybrid a versatile microrobot at the nanoscale that can effectively release its cargo by environmentally inherent or noninvasive on-demand external cues.

### Guiding bacterial biohybrids magnetically toward tumor spheroids

**Fig. 3.**
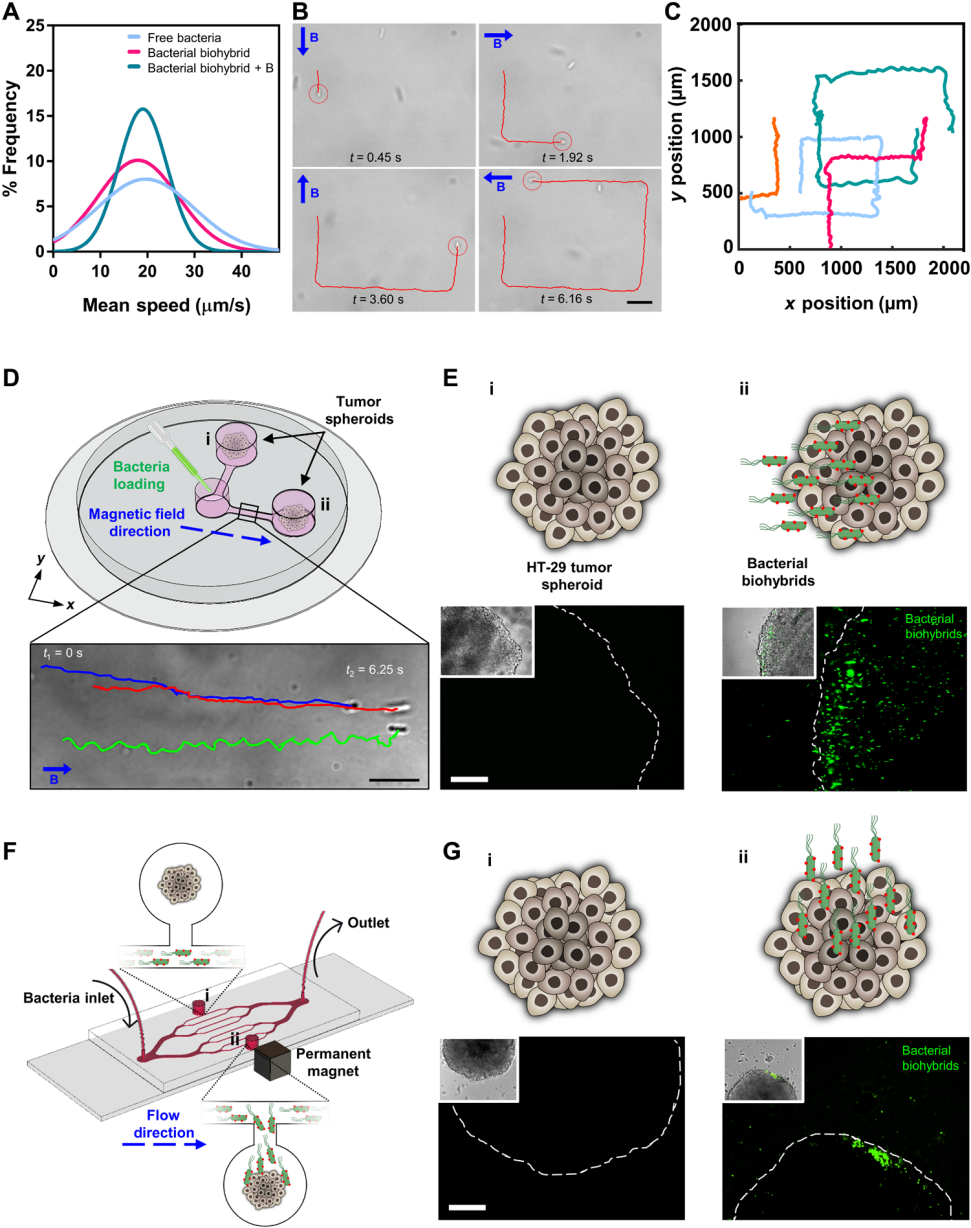
Motility characterization, external magnetic control, and tumor spheroid localization of magnetic bacterial biohybrids. (**A**) 2D swimming velocity analyses of free bacteria and bacterial biohybrids with or without applied external magnetic field. (**B**) Magnetic control of bacterial biohybrids by changing the applied field (10 mT) direction by 90° turns. B represents the magnetic field vector. Scale bar, 10 μm. (**C**) 2D swimming trajectories of bacterial biohybrids under external magnetic field control. (**D**) A magnetic guidance setup with three reservoirs, two of which contain tumor spheroids (i and ii), connected by narrow channels to the loading reservoir. A uniform magnetic field (26 mT) along the *x* axis was created with a permanent magnet setup. Scale bar, 10 μm. (**E**) Schematics and microscopy images of the tumor spheroids in reservoirs i and ii. Scale bar, 100 μm. (**F**) A microfluidic system with branched channels and two reservoirs (i and ii) with one tumor spheroid in each. A small permanent magnet was placed next to the reservoir ii to generate a magnetic field gradient. (**G**) Schematics and microscopy images of the tumor spheroids in reservoirs i and ii. Scale bar, 100 μm.

Swimming motility of bacterial biohybrids was characterized and compared to that of free bacteria after the conjugation of mNPs (Fig. 3A). An average swimming velocity of free bacteria was recorded as 19.9 ± 9.3 μm/s, whereas for magnetic bacterial biohybrids, swimming speed was 17.4 ± 6.5 μm/s. We performed the same analysis with a continuously applied magnetic field (10 mT) by demonstrating a square-shaped swimming path (Fig. 3, B and C, and movie S2) and measured the average swimming velocity at 18.5 ± 4.7 μm/s, which is comparable with the average velocity of both free bacteria and bacterial biohybrids without applied magnetic fields. Furthermore, we applied different external magnetic field strengths (5, 10, and 20 mT) to bacterial biohybrids to investigate whether the field strength has an impact on motility. Swimming velocities were recorded at 16.3 ± 4.1, 18.5 ± 4.7, 16.4 ± 1.7, and 17.7 ± 2 μm/s for the applied magnetic field of 5, 10, 15, and 20 mT, respectively (fig. S5). This indicates that externally applied magnetic fields or the field strength do not have a notable impact on the motility of bacterial biohybrids.

To demonstrate the facile and effective external control potential of bacterial biohybrids migrating toward a solid tumor, we performed magnetic guidance experiments with colon tumor spheroids integrated within closed microchannels (Fig. 3D). The magnetic guidance setup consists of two orthogonal microchannels with two reservoirs at each end (reservoirs i and ii; with one tumor spheroid in each) and connecting to a third loading reservoir. The microchannel platform was placed in between two permanent magnets that create a uniform magnetic field (26 mT) parallel to one of the channels (Fig. 3D, *x* axis) and vertical to the other one. Once the bacterial biohybrids were loaded into the middle reservoir, mNPs on the biohybrids align themselves with the uniform magnetic field in the *x* axis; thus, bacterial biohybrids start swimming parallel to the applied magnetic field toward the tumor spheroid in reservoir ii (movie S3). After 30 min of continuous magnetic guidance toward the spheroid in reservoir ii, fluorescence microscope images were taken of both spheroids, where green color represents GFP-expressing bacterial biohybrids (Fig. 3E). Microscopy images showed that there were no biohybrids around the tumor spheroid in reservoir i (Fig. 3E), because the applied magnetic field did not direct the bacterial motion upward (*y* axis), whereas tumor spheroid in reservoir ii was accumulated with bacterial biohybrids (Fig. 3E).

While magnetic steering of bacterial biohybrids is useful for controlling their trajectory in local microenvironments, their accumulation into a specific microenvironment needs to be facilitated (e.g., localization of biohybrids in peripheral tissues introduced through blood circulation), which could be achieved by using magnetic gradients. To further test the magnetically induced accumulation of bacterial biohybrids under flow conditions, a branched microchannel mimicking blood vasculature was designed, where bacterial biohybrids were continuously flown through the branches at a mean flow rate of 15.625 μm/s (Fig. 3F). Two small reservoirs (i and ii) were connected to the outermost lateral branches, and one tumor spheroid was placed in each. A cubical permanent magnet was attached next to the reservoir ii, which generated a sufficient magnetic field gradient to attract continuously flown bacterial biohybrids toward the tumor spheroid in reservoir ii (Fig. 3F, fig. S6, and movie S4). Fluorescence images demonstrated that after 1 hour of continuous flow and applied magnetic field gradients, there were no bacterial biohybrids at the vicinity of the tumor spheroid in reservoir i due to the lack of any magnetic gradients influencing the upper reservoir, whereas tumor spheroid in reservoir ii was accumulated with bacterial biohybrids (Fig. 3G). With the biohybrid design presented here, we were not only able to preserve the inherent swimming velocity and motility of bacteria, but we also actively guided bacterial biohybrids using various forms of magnetic fields and flow conditions and showed the colonization of tumor spheroids, which might prove to be an essential aspect for localized cargo delivery applications.

### Enhanced penetration into 3D biological matrices under magnetic fields

**Fig. 4.**
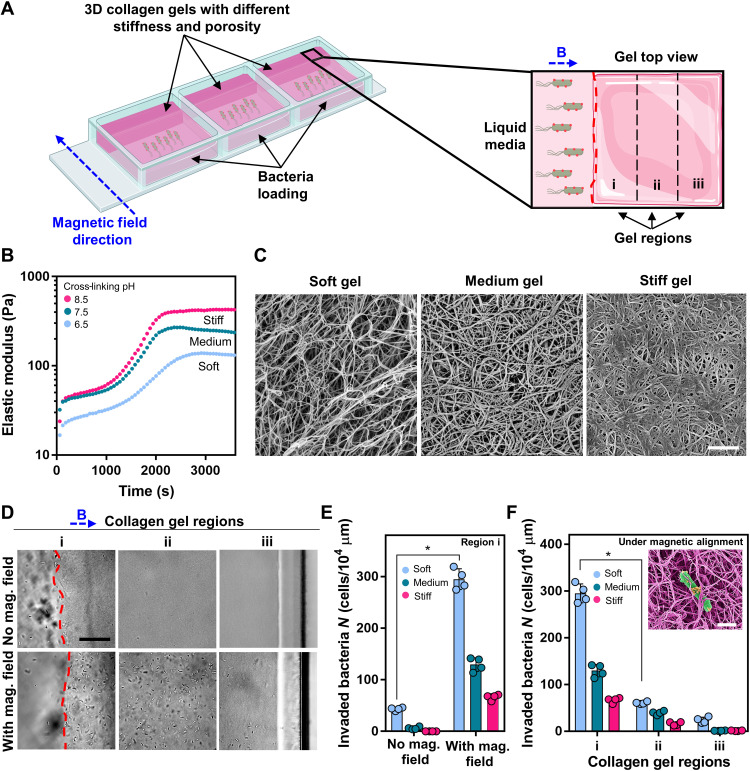
Invasion of collagen gels by magnetically aligned bacterial biohybrids. (**A**) Schematic of the experimental setup demonstrating 3D collagen gels with different stiffnesses cross-linked on one side of a tissue culture plate. A permanent magnet setup generating a uniform magnetic field (26 mT), directing bacterial biohybrids toward the collagen gel continuously. (**B**) Dynamics of collagen gel cross-linking at 37°C. (**C**) SEM images of 3D collagen gels of varying cross-linking pH. Scale bar, 1 μm. (**D**) Bright-field optical microscopy images of collagen gel regions after overnight incubation with bacterial biohybrids, with and without magnetic field. Gels were divided into three regions: i, ii, and iii. The red dashed line represents liquid-gel interphase. Gels represented in images were prepared at pH 7.5. Scale bar, 50 μm. (**E**) Quantification of bacterial biohybrids within three different collagen gels after overnight incubation with or without magnetic field (Student’s t test, *P* < 0.05). Error bars represent the SD of the mean. (**F**) Quantification of bacterial biohybrids within three different collagen gels after overnight incubation under uniform magnetic field (Student’s t test, P < 0.05). Error bars represent the SD of the mean. Inset shows a pseudocolored SEM image of a bacterial biohybrid embedded in collagen gel. Scale bar, 1 μm.

Bacterial motion in liquid environments, including biological media, is well studied and understood (*13*, *35*). While the motion of bacterial biohybrids in biological liquid media is relevant to their medical applications, their motility and performance in viscoelastic and solid environments, such as biological gels and extracellular matrix (ECM), are also crucial for reaching deep organs and tumor tissues. To test and demonstrate the tissue penetration and invasion capability of our bacterial biohybrids into the local ECM of healthy and tumor tissues, we performed 3D matrix invasion experiments with bacterial biohybrids under constant magnetic field alignment (Fig. 4A).

We prepared a series of collagen gels with different stiffness and morphology and placed them on one side of a well plate (Fig. 4A). Collagen gel stiffness and porosity were varied by changing the cross-linking pH (pH 6.5, 7.5, and 8.5), while all the other parameters (i.e., collagen concentration, cross-linking temperature, and ionic strength) were kept constant. Rheology characterizations showed that gels became stiffer as the pH of the polymerization solution increased, with higher equilibrium storage modulus recorded at pH 8.5 condition (425 Pa) than pH 7.5 (236 Pa) and pH 6.5 (131 Pa) conditions (Fig. 4B), which is in agreement with the fact that all metrics for collagen gel moduli increase with increasing pH of the polymerization solution (*36*). Consequently, collagen gels polymerized at pH 6.5 were called “soft” gels, and those polymerized at pH 7.5 and pH 8.5 were called “medium” and “stiff” gels, respectively. In addition to rheological characterizations, we performed SEM imaging to explore the morphology and the porosity of various collagen gels (Fig. 4C). We observed defined variations in porosity and the density of fiber bundles and an inverse correlation of porosity with gel stiffness. Compared to medium and stiff gels, soft gels not only held a lower elastic storage modulus but also presented larger pore sizes ranging from nano- to micrometer (Fig. 4C). By preparing 3D gels with varying stiffness and porosity, we sought to explore the bacterial navigation in confined media that are common in many extracellular tissue matrices.

After soft, medium, and stiff gels were cross-linked on the side, the rest of the wells were filled with cell culture media. 3D gels took one-third of the well surface area, and gel height was always kept higher than the cell culture medium height to prevent transport of bacterial biohybrids to the top of the gels through the media. Then, bacterial biohybrids were pipetted at the far end of the liquid side of the wells and incubated overnight under constant uniform magnetic field (26 mT) alignment for collagen gel invasion (Fig. 4A). We divided the collagen gels into three distinct regions (i, ii, and iii) where each region corresponds to a cross-sectional area of 9.4 mm^2^ (Fig. 4A). Another well plate with soft, medium, and stiff gels and bacterial biohybrids was incubated overnight without any magnetic field as a control experiment. Microscopy images of collagen gel regions were taken after overnight incubation of the setup (Fig. 4D and fig. S7). From the microscopy images, we observed that a minimum amount of bacterial biohybrids were able to pass through the liquid-gel interface and reach region i without magnetic alignment, whereas a large population of bacterial biohybrids could pass the interface and easily penetrate up to the end of the well (region iii) when incubated overnight under aligning magnetic fields. In region i, we observed a 7-fold increase in invaded bacterial biohybrid number inside the soft gel and a 22-fold increase for the medium gel when there was a magnet directing microswimmer motion toward the gel (Fig. 4E). There were no bacterial biohybrids detected at region i of the stiff gel without magnetic fields, but approximately 70 bacterial biohybrids per 10^4^ μm^2^ were detected at region i of the stiff gel under guiding magnetic fields (Fig. 4E).

We further quantified the invaded bacteria density at different regions of all three gel types under magnetic fields to investigate the role of gel stiffness and porosity on tissue penetration. As expected, the number of invaded bacterial biohybrids decreased in general as the collagen gel stiffness increased and porosity decreased (Fig. 4F). A 5-fold decrease was observed for the soft gel and a 3.4-fold decrease was observed for both medium and stiff collagen gels when comparing the number of invaded bacterial biohybrids in regions i and ii. There were only a few bacterial biohybrids detected at region iii for all three gel conditions; soft gel had around 25 bacterial biohybrids per 10^4^ μm^2^, whereas medium and stiff gels were invaded by around 11 and 12 bacteria per 10^5^ μm^2^, respectively (fig. S7). Although the exact mechanisms of bacterial penetration and navigation within such 3D biological matrices still remain unclear (*37*), our results indicate that bacterial biohybrids, in addition to their run and tumble motion, get trapped inside porous structures (Fig. 4F, inset) and jump through them to navigate (movie S5). The results reported here demonstrated that bacterial biohybrids could penetrate and move inside a confined and porous biological microenvironment under constant magnetic alignment. Furthermore, we demonstrated that the gel stiffness and porosity dictate bacterial colonization within a confined space, with more bacterial biohybrids penetrating and accumulating within soft and porous collagen gels compared to stiffer and less porous gels.

### Bacterial biohybrid-mediated on demand drug delivery to tumor spheroids

We engineered a photothermally active bacterial biohybrid design to show the on-demand, NIR-triggered release of therapeutic agents in 3D tumor spheroids. Light-triggered drug delivery mechanisms offer the possibility of therapeutic release under a spatiotemporally optimum condition, minimizing unwanted leakage of molecules, thus achieving highly effective therapeutic benefits compared to passive systems. By combining stimuli-responsive drug release mechanisms with bacterial biohybrids, we aimed to preserve a high amount of therapeutics loaded on the biohybrids and concentrate the biohybrids around the vicinity of tumor spheroids until NIR stimulus to maximize therapeutic transport.

**Fig. 5.**
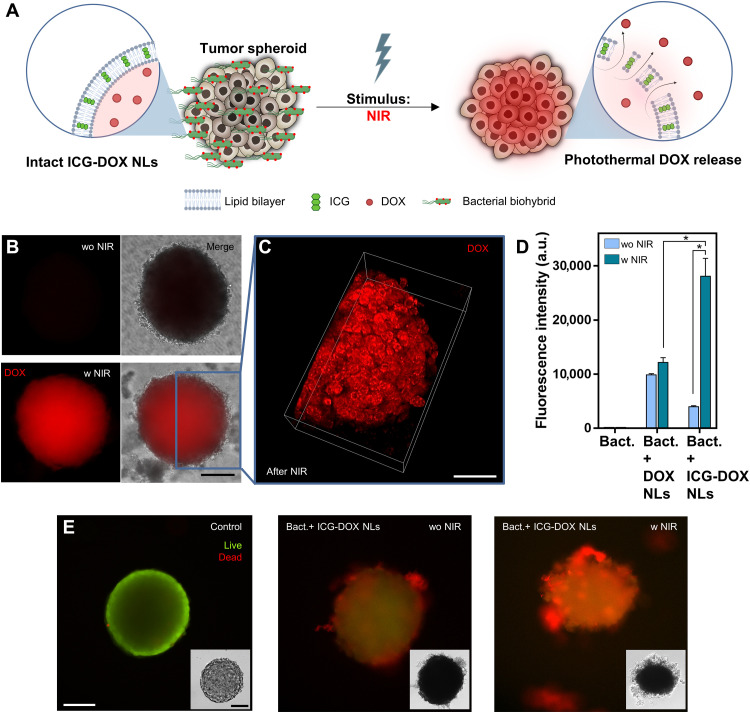
NIR-driven drug release from bacterial biohybrids and drug uptake by tumor spheroids. (**A**) Bacterial biohybrids carrying stimuli-responsive ICG-DOX NLs are localized on tumor spheroids and release their payload upon NIR stimulus. NLs convert the light energy into heat, which then prompts phospholipid disintegration and ultimately triggers the release of DOX molecules. (**B**) DOX delivery to HT-29 tumor spheroids was achieved by coculturing the spheroids with bacterial biohybrids and irradiating the coculture setup with NIR light (~0.6 W/cm^2^, 10 min). Fluorescence microscopy images show DOX signal from the spheroid 24 hours after NIR irradiation. Scale bar, 100 μm. (**C**) 3D view of confocal microscopy image shows DOX uptake within the cells of the tumor spheroid 24 hours after NIR irradiation. Scale bar, 75 μm. (**D**) Fluorescence intensities of free bacteria, bacterial biohybrids carrying DOX NLs, and bacterial biohybrids carrying ICG-DOX NLs, with or without NIR irradiation (Student’s *t* test, *P* < 0.05). Error bars represent the SD of the mean. (**E**) Live/dead viability staining of HT-29 tumor spheroids with bacterial biohybrids carrying ICG-DOX NLs, with and without NIR irradiation. Green and red colors indicate live and dead cells, respectively. Insets show bright-field images of the spheroids. w, with; wo, without. Scale bars, 100 μm.

We prepared 3D spheroids with HT-29 colon cancer cells and checked the spheroid viability after various durations of NIR irradiation (5, 10, and 15 min) to determine any possible adverse effects that might originate from light irradiation. All spheroids demonstrated high viability (97.6 ± 6.1%, 95.3 ± 5.0%, and 93.5 ± 6.2% for 5-, 10-, and 15-min irradiation, respectively) after NIR irradiations (fig. S8), and we continued on-demand release experiments with 10-min NIR irradiation. Each of the free bacteria and bacterial biohybrids carrying DOX NLs, ICG NLs, or ICG-DOX NLs was incubated with tumor spheroids for 10 min before NIR light application. Bacterial cells are known to accumulate around the vicinity of tumor tissues in vivo (*15*) and tumor spheroids in vitro (*25*, *38*), which is in agreement with our experimental observations showing large numbers of bacteria concentrating on the HT-29 tumor spheroids when coincubated for 10 min (movie S6). After localization of bacterial biohybrids on tumor spheroids, we irradiated the samples with NIR to photothermally activate the liposomal drug delivery platform, where NIR light is absorbed, and the absorbed energy is converted to heat, causing a temperature increase (Fig. 5A). After NIR-triggered drug release, samples were incubated for 24 hours and fluorescently imaged for DOX uptake (excitation, 470 nm; emission, 585 nm) by tumor spheroids (fig. S9). Tumor spheroids coincubated with bacterial biohybrids carrying stimuli-responsive NLs demonstrated a substantial amount of fluorescence signal 24 hours after NIR irradiation, whereas a negligible fluorescent signal was observed on spheroids without NIR irradiation (Fig. 5B). Confocal fluorescence microscopy further confirmed the uptake of DOX molecules by tumor spheroids after NIR trigger (Fig. 5C). We further quantified the fluorescence intensity of each spheroid for different conditions with or without NIR irradiation (Fig. 5D). DOX uptake was increased by sevenfold when NIR light was applied to bacterial biohybrids carrying stimuli-responsive NLs. A minimum amount of DOX uptake by tumor spheroids was observed for some samples even when there was no NIR trigger, which could be attributed to the release of DOX through simple diffusion or pH-based release due to the pH gradients found on HT-29 tumor spheroids (*39*). In the case of no NIR irradiation, DOX uptake by tumor spheroids was slightly higher with biohybrids carrying only DOX NLs, compared to biohybrids carrying stimuli-responsive NLs. This is most likely due to increased membrane stability of stimuli-responsive NLs, because the addition of lipophilic molecules (e.g., ICG) to phospholipid bilayers increases the stability of the lipid membrane and minimizes the leakage of hydrophilic molecules, including DOX (*40*). We further tested the cancer cell-killing efficiency of bacterial biohybrids by coincubating them with 3D tumor spheroids with and without NIR treatment (Fig. 5E). Compared to the control group, bacterial biohybrids resulted in death of cancer cells in spheroids, which was further enhanced under NIR irradiation (Fig. 5E). With stimuli-responsive and photothermally activated liposomal drug delivery mechanism, we demonstrated localized and external stimuli-triggered on-demand release of anticancer therapeutics, demonstrating an active delivery platform with bacterial biohybrids.

## DISCUSSION

To achieve desired minimally invasive cargo delivery applications with biohybrid microrobots, the design constructs should have high-throughput fabrication, efficient motility, tissue penetration capability, multifunctional operation, and stimuli-responsive external control (including steering and triggered cargo release). Many bacterial biohybrid microrobot designs suffer from low to average conjugation yield, with only a small fraction of cells carrying an artificial cargo. In particular, because bacteria have short doubling times (e.g., 20 min for wild-type *E. coli* under optimal conditions) (*13*), some of the conjugated cargoes will be eventually lost during the growth phase, which causes a loss of payload efficiency and weakening the magnetic steerability over long time periods. Therefore, a high-throughput conjugation process and characterization of time window are required for efficient magnetic steering in medical implementations. Aside from throughput, the conjugation approach should not have an adverse effect on either the bacteria or the functional properties of artificial carriers. The method of conjugation shown here, i.e., streptavidin-biotin complex, did not require any extreme reactions or incubation processes, thus assuring a durable and functioning bacterial biohybrid without sacrificing any of the intrinsic capabilities, including motility and penetration capability of bacteria. Furthermore, by using nanometer-size cargoes as the artificial units of the biohybrid design, i.e., mNPs and multifunctional, stimuli-responsive NLs, we preserved the simplicity and motility of the final construct that can be actively controlled.

Bacterial motility is a crucial aspect for the guidance of biohybrid microrobots both by local chemical and pH gradients as well as external steering mechanisms, such as magnetic fields. The intrinsic swimming ability of bacteria supports their capability to sense their surroundings based on the diffusion of chemoattractants, causing biased movement (*13*), which was previously exploited to chemically control bacterial biohybrids (*41*). External control mechanisms, including magnetic fields, on the other hand, allow more precise and controlled guidance compared to chemotaxis-based motion of the biohybrid microrobots. Therefore, external magnetic fields are extensively used in the literature to control the swimming motion of magnetic bacterial biohybrids (*10*, *25*). Bacterial motility is also critical for penetration and distribution within the tissue microenvironment, such as solid tumors. Several studies have shown that highly motile bacterial strains can overcome penetration limitations and can accumulate tumor tissues more effectively compared to less motile strains (*42*–*44*). Considering the importance of the bacteria motility on sensing, guided motion, and tissue penetration, a biohybrid platform should preserve the inherent swimming characteristics of the bacterial unit. Along with a harsh conjugation process, size and compatibility of the artificial unit can severely hamper the bacterial motility and thus affect bacterial microrobot performance. Many biohybrid microrobotic designs reported in the literature lack the extensive characterization of swimming and controllability after the insertion of the artificial units to microorganisms. After the conjugations, the bacterial biohybrid design reported here maintained the original swimming characteristics and speed, which is higher than previously reported swimming speeds for bacterial biohybrids decorated with similar-size artificial units (*19*, *45*, *46*).

Biohybrid microswimmers were previously shown to transport drug molecules and kill cancer cells in both 2D monolayer and 3D spheroid tumor models (*9*, *11*, *16*, *47*), although most platforms mainly relied on diffusion-based release once the swimmers reached their target. On the other hand, stimuli-responsive cargo delivery systems offer spatiotemporal control over the release of molecules in a localized and on-demand manner. In this work, we prepared multifunctional and stimuli-responsive liposomal drug release platform for efficient release of molecules from bacterial biohybrids. We integrated two stimuli-responsive release mechanisms to achieve a multimodal and robust drug release system that can be activated via either external, noninvasive cues (NIR) or internal, microenvironment-specific conditions (low pH). Light-triggered drug release mechanisms are attractive due to their stimuli responsiveness and efficient spatiotemporal control over cargo delivery. With light-triggered release mechanisms, the cargo is maintained on the carrier until the target location is reached and released on the spot, preventing undesired leakage due to diffusion or burst release. Moreover, among different light sources, NIR, with an emission wavelength of 700 to 900 nm, is one of the safest drug release stimuli with deeper tissue penetration and lower cellular cytotoxicity (*48*). Furthermore, higher temperatures and penetration depths can be achieved by more advanced (e.g., pulsed) and powerful NIR laser sources at different wavelengths along with a higher concentration of photothermal agent loading into the NLs. In addition, the pH-responsive nature of our liposomal formulation offers higher drug release at low pH due to protonation of the carboxyl groups at the lipid bilayer and subsequent phase transition and disruption of the membrane (*49*). Thus, the biohybrid microrobot platform presented here is capable of drug release multimodally, i.e., photothermal release and pH-driven release, that can be triggered with both pathophysiological changes in the local microenvironment, such as low pH in solid tumors, and external fields.

Since swimming bacteria were first found in the 17th century (*13*), bacterial motility in liquid media has been widely studied, whereas in physiological settings, most bacteria must steer the obstacles, overcome viscous forces, and find pores to squeeze through solid tumors, tissues, mucosa, and biological gels. However, there is still a limited understanding of how bacteria move in such confined matrices and how they penetrate tumor tissues (*37*). We prepared 3D collagen gels as tissue scaffolds using collagen type I, which is the most prevalent form of collagen that closely mimics in vivo tumor ECM conditions (*50*). In our experiments, we observed nonmonotonous motion of bacterial biohybrids inside 3D gels, where they got trapped within the pores until they were released and hopped to the next one. In addition, when the biohybrid motion is guided under external magnetic fields, persistent motion of the microrobots along the magnetic field and toward the gel enabled a larger population to invade further into the porous biological matrix. While our results indicate enhanced penetration of bacterial biohybrids under magnetic fields, the higher density and hierarchical structure of the ECM in real tumors could pose additional challenges for bacterial penetration into tumors. Such challenges can be overcome by modification of bacterial biohybrids with ECM-degrading enzymes (e.g., collagenase). In addition, studying the exact swimming mechanisms in porous and viscous media with various tools, such as 3D biological gels and perfused tumor tissues, would be the next step to provide a fundamental understanding of bacterial navigation within such confined spaces. Such mechanistic understanding of bacterial motility in confined spaces, along with artificial taxis capabilities, such as magnetic guiding as shown in our study, could create more possibilities for effective penetration and transport of anticancer therapies to solid tumors.

Aside from biohybrid microswimmers, wild-type and genetically engineered bacteria have also been used for more than a hundred years for cancer therapy due to their specific targeting of tumor necrotic regions and activation of antitumor immune response in the host body (*51*, *52*). However, the degree of immune stimulation needs monitoring, because overstimulation may also cause other local or systemic toxicity and off-target tissue damage (*15*). Therefore, both in bacteria-mediated and biohybrid cancer therapies, it is essential to establish an optimum balance between the immune stimulation and the safety of the therapy and to provide a “safety” tool, which could be achieved by either genetically engineering the bacteria or biohybridizing with immune compatibility. In the literature, various genetic engineering approaches have been used to improve the safety of bacterial therapies, such as attenuation of pathogenic bacterial species [including various *Salmonella* (*53*–*55*) and *E. coli* strains (*56*, *57*)], termination switches (*14*, *58*, *59*), and immune evasion (*60*). Current bacteria-mediated cancer therapy clinical trials (*14*) include *Salmonella typhimurium* VNP20009 (NCT01099631), an attenuated strain of *Salmonella* to improve the safety of therapy; SYNB1891 (NCT04167137), a probiotic strain of *E. coli* Nissle engineered to synthesize Stimulator of Interferon Genes (STING) agonist cyclic di-adenosine monophosphate; and *Listeria monocytogenes* (NCT02325557), which is commonly used to express and secrete antigen-adjuvant fusion proteins.

Despite the great potential of genetically engineered bacterial therapies, the cost-intensive nature of the approach along with long-term safety-related concerns hampers their translation to the clinic. On the other hand, biohybrid approaches offer cost-effective means to embark bacteria with advanced functionalities, such as magnetic manipulation and drug loading, which is quite challenging to achieve through genetic engineering approaches. In addition, alternative to the bioengineering, termination switches (*10*) and immune-evasion properties (*20*) can be also added on engineered synthetic cargoes. In our bacterial biohybrid design, liposome membranes could be further modified to include immune-compatible molecules (e.g., zwitterionic formulations, CD47—“do not eat me signal”) for modulating the immune response. In addition, NIR-triggered heating mechanism could also be used as a termination switch for the bacteria, as bacteria viability decreases at increased temperatures (*10*). Furthermore, proliferation of bacteria inside the human body could be limited by controlled administration of antibiotics (*61*).

Our design strategy establishes an advanced and optimized fabrication route for bacteria-based biohybrid microrobots with exceptional performance and multifunctionality, together with (i) maintained motility of bacterial biohybrids after functionalization, (ii) 3D matrix penetration capabilities, and (iii) stimuli-responsive cargo delivery. The biohybrid design reported here presents a highly efficient assembly route for a magnetically controlled biohybrid microrobotic system with swimming velocities far higher than previous biohybrid designs incorporating *E. coli*. The retained motility allowed for dynamic navigation control with externally applied magnetic fields, which aligns and guides the bacterial biohybrids through 3D porous microenvironments and localizes them in tumor tissues. Photothermal and pH-sensitive liposomal carriers on biohybrids display a versatile and on-demand delivery platform that is activated through NIR light and pH, allowing the release of chemotherapeutic molecules spatiotemporally at the target. The design approach introduced here showcases an efficient microrobotic platform with motile bacteria to overcome physiological barriers for multifunctional and stimuli-responsive cargo delivery.

## MATERIALS AND METHODS

### Bacteria culture

A genetically engineered strain of *E. coli* MG1655 was provided by Victor Sourjik laboratory at the Max Planck Institute for Terrestrial Microbiology. Cells were initially cultured on tryptone agar plates [10 g of tryptone (Santa Cruz Biotechnology Inc.), 5 g of NaCl (Merck), and 15 g of agar-agar (VWR Chemicals) in 1 liter of distilled water]. Single colonies were transferred to 5 ml of tryptone broth (TB) medium (10 g of tryptone and 5 g of NaCl, in 1 liter of distilled water, pH adjusted to 7.0) supplemented with kanamycin (50 μg/ml; Merck), ampicillin (100 μg/ml; Merck), and 1 μM biotin (Merck) for overnight culture at 37°C and 200 rpm. Next, 100 μl of the overnight culture was transferred to 10 ml of TB medium supplemented with 1 μM biotin and cultured for 2 hours at 34°C and 270 rpm. Then, 250 μM IPTG (Merck) and *L*-(+)-arabinose (10 μg/ml) were added to bacteria culture, which was further cultured at 34°C and 270 rpm until an optical density at 600 nm (OD_600_) of 0.5 to 0.6 is reached as measured by a plate reader (BioTek Synergy H1).

### Preparation of free NLs

A well-established thin-film hydration and extrusion method was used for the preparation of various biotinylated liposome formulations. 1,2-Distearoyl-*sn*-glycero-3-phosphocholine [18:0 PC (DSPC)], 1,2-distearoyl-*sn*-glycero-3-phosphoethanolamine-*N*-[methoxy(polyethylene glycol)-2000] (18:0 PEG2000 PE), 1,2-distearoyl-*sn*-glycero-3-phosphoethanolamine-*N*-[biotinyl(polyethylene glycol)-2000] [DSPE-PEG(2000) Biotin], and cholesterol were used at 60:4:1:35 molar ratio. Addition of cholesterol increases the structural integrity of NLs and helps prevent their fusion with the bacterial cell membrane. Octadecylamine was added to the lipid mixture to prepare cationic liposomes. For flow cytometry and fluorescence microscopy experiments, 0.5 mole percent of 1,2-distearoyl-*sn*-glycero-3-phosphoethanolamine-*N*-(cyanine 5) (18:0 Cy5 PE) was added to lipid mixture. All lipids are purchased from Avanti Polar Lipids. First, all lipids were mixed in chloroform (Merck) at a predetermined molar ratio in a round-bottomed flask. Next, chloroform was evaporated using a rotary evaporator (Heidolph Instruments) at 40°C and 250 rpm for 2 hours, and then the flask was left under high vacuum overnight to remove any remaining solvent traces. Thin lipid film was hydrated with phosphate-buffered saline (PBS) (1×, pH 7.4) to a final concentration of 1.56 mg/ml at 55°C and 300 rpm for 2 hours in a temperature-controlled shaker. Next, by using a mini-extruder (Avanti Polar Lipids, AL), multilamellar vesicles were sized down by passing them through a set of polytetrafluoroethylene (PTFE) membrane filters with pore sizes of 1, 0.4, and 0.2 μm. Lipid suspension was passed through each membrane filter 15 times to obtain NLs with 200 nm diameter. NLs were stored at 4°C until further use.

### Preparation of DOX- and ICG-loaded NLs

DOX (Sigma-Aldrich) loading was achieved in an ammonium sulfate gradient by remote loading method. After evaporation of chloroform, thin lipid film was hydrated with aqueous ammonium sulfate solution (250 mM, pH 5.5) in a temperature-controlled shaker at 55°C and 300 rpm for 2 hours. After hydration, multilamellar vesicles were extruded through PTFE membrane filters. Then, NL solution was passed through a PD-10 desalting column (Cytiva) packed with Sephadex G-25 resin and preequilibrated with PBS (1×, pH 7.4) to exchange the ammonium sulfate outside of the NLs with PBS, creating a transmembrane pH gradient. DOX solution (1 mg/ml; in PBS) was then introduced to NL suspension and incubated in a temperature-controlled shaker at 60°C and 300 rpm for 1.5 hours. Subsequently, unencapsulated DOX was removed by passing through a PD-10 desalting column preequilibrated with PBS (1×, pH 7.4). ICG was incorporated into the phospholipid bilayer of liposomes by adding ICG (1 mg/ml; in methanol) at various ICG:phospholipid molar ratios (1:625, 1:250, 1:100, and 1:75) to the lipid mixture before the evaporation step. Unloaded ICG was removed after the extrusion step by passing the NL suspension through a PD-10 desalting column preequilibrated with PBS (1×, pH 7.4). NLs loaded with both DOX and ICG (ICG-DOX NLs) were prepared by encapsulating DOX to ICG-containing NLs by remote loading method. For photothermal characterizations, the ICG concentration in free ICG sample was 1.3 μM (1 mg/ml), whereas the raw ICG concentrations in different ICG:phospholipid nanoliposomal formulations were 0.032 μM (1:625), 0.08 μM (1:250), 0.2 μM (1:100), and 0.266 μM (1:75). For further experiments with ICG NLs and ICG-DOX NLs, liposomes were prepared with 1:100 ICG:phospholipid molar ratio, which corresponds to 0.2 μM ICG in NL solution (1.56 mg/ml). NLs were stored at 4°C until further use.

### Characterization of NLs

All NL suspensions were separately characterized by DLS (Möbius, Wyatt Technologies) for size and zeta potential measurements and ultraviolet (UV)/visible/NIR spectroscopy (Lambda 1050, PerkinElmer Inc.) for absorbance readings after DOX and ICG loading steps. For both measurements, the NL suspensions were diluted to 0.1 mg/ml in distilled water, and suspensions were then placed in measurement cuvettes. Hydrodynamic radii, zeta potentials, and absorbance spectra were measured successively three times, and an average of the measurements was reported. The amount of encapsulated DOX was characterized by measuring DOX fluorescence signal after lysis of NLs. Briefly, DOX-loaded and washed NL solution was treated with aqueous Triton X solution (4%, v/v) for 15 min and centrifuged at 30,000*g* for 30 min. The supernatant fluorescence was measured with a plate reader (excitation, 485 nm; emission, 528 nm). A standard curve was created by measuring the fluorescence of DOX solutions prepared at different concentrations. DOX loading efficiency was calculated using the following equation

$$\text{EE}\%=\frac{\text{Encapsulated DOX concentration}}{\text{Total DOX concentration}}\times 100$$

### Tissue phantom preparation

For photothermal conversion experiments through a tissue mimic, a scattering and absorbing agar phantom was prepared with Intralipid 20% emulsion (Sigma-Aldrich) and black ink (Pelikan) as the scattering and absorbing components, respectively. Briefly, 0.45 g of agar (VWR Chemicals) was dissolved in 30 ml of dH_2_O and heated in the microwave until boiling. Intralipid 20% emulsion (0.62 ml) was heated in a hot water bath. Heated Intralipid 20% emulsion was added to hot agar solution and gently mixed. Ink (0.6 ml of 1:100 diluted stock in dH_2_O) was added to the agar and Intralipid mixture and gently swirled. This solution (7.8 ml) was poured into a petri dish. The solution was cooled at room temperature for 30 min to solidify and form a 1-mm-thick phantom, which was used immediately.

### NIR-mediated heating of ICG NLs and ICG-DOX NLs

PBS (1×), ICG solution (1.3 μM; in methanol), and ICG NLs composed of various ICG:phospholipid molar ratios (1:625, 1:250, 1:100, and 1:75) were loaded in capillary glass tubes and irradiated with a NIR laser (808 nm, 0.6 W/cm^2^) with or without agar tissue phantoms. Thermal images were obtained, and temperature information was recorded with an infrared thermal camera (ETS320, FLIR Systems).

### pH- and temperature-responsive DOX release

To characterize pH-responsive DOX release, DOX NLs (0.5 ml, 3.16 mg/ml) were loaded in dialysis devices (Slide A Lyzer MINI Dialysis Device, 10K MWCO). Next, dialysis devices were inserted into 15-ml conical tubes filled with dialysis buffer (PBS; 14 ml) with various pH (7.4, 6.5, 5.5, and 2.5), which were then placed in a temperature-controlled shaker at 37°C and 150 rpm. At predetermined time intervals, samples were collected from dialysis buffers and replaced with the equal volume of fresh buffer. DOX release was quantified from the collected buffer samples by measuring the fluorescence with a plate reader at excitation of 485 nm and emission of 528 nm. NIR-triggered DOX release was characterized by irradiating ICG-DOX NLs with a NIR laser (808 nm, 0.6 W/cm^2^) for 5 min and then loading dialysis buffer samples into dialysis devices at predetermined time intervals as they were kept at 37°C and 150 rpm shaking inside a shaker. As a control, ICG-DOX NLs were kept at fixed temperatures of 37°, 43°, and 55°C for 5 min, and all samples were then further kept at 37°C and 150 rpm inside a temperature-controlled shaker. Samples from the dialysis buffer were collected at predetermined time intervals. All sample collections and fluorescence measurements were performed as previously described.

### Preparation of bacterial biohybrids carrying mNPs and NLs

Bacterial biohybrids were prepared by the streptavidin-biotin interaction between a genetically engineered strain of *E. coli* MG1655 with GFP and biotin expression and streptavidin-functionalized mNPs (micromod Partikeltechnologie GmbH). First, bacterial cells were rinsed with motility medium [10 mM K_2_HPO_4_, 10 mM KHPO_4_, 67 mM NaCl, 0.1 mM EDTA, and 1% (w/v) glucose (pH 7.0)] after OD_600_ of 0.5 to 0.6 was reached as previously described. mNPs were suspended in Dulbecco’s PBS (Sigma-Aldrich) at a concentration of 3.6 mg/ml and mixed with bacterial cells suspended in motility medium in a microtube with a final particle concentration of 0.36 mg/ml. The mixture was incubated for 1 hour at 34°C and 270 rpm in a temperature-controlled incubator. After incubation, bacterial biohybrids were washed three times with fresh motility media by centrifugation at 2000*g* for 5 min. For further conjugation of NLs to bacterial biohybrids, a three-step approach was followed. First, bacterial cells were coated with mNPs, then bacterial biohybrid surface was treated with streptavidin to cover free biotin groups, and, as a final step, biotinylated NLs were directly conjugated, forming a biotin-streptavidin-biotin system. This triple biotin-streptavidin-biotin binding complex, along with the PEG group on the NLs, functions as a stable spacer, preventing the fusion of synthetic cargoes with the bacterial cell membrane. Briefly, bacterial biohybrids were incubated with streptavidin (100 μg/ml; Sigma-Aldrich) for 30 min at 34°C and 270 rpm, washed twice with fresh PBS, and further incubated with biotinylated NLs (1.56 mg/ml) for 1 hour at 34°C and 270 rpm. After incubation, bacterial biohybrids carrying mNPs and NLs were washed three times and suspended in fresh motility media for further use. An mCherry- and biotin-expressing *E. coli* MG1655 strain was also used for the construction of biohybrid microswimmers with mNPs and NLs (movie S7).

### Characterization of bacterial biohybrids

Bacterial biohybrids were characterized by fluorescence imaging, flow cytometry, and SEM imaging. For fluorescence imaging of free bacteria, a genetically engineered GFP-expressing *E. coli* strain was used. 18:0 Cy5 PE (excitation, 646 nm; emission, 663 nm) and mNPs with red fluorescence (excitation, 552 nm; emission, 580 nm) were used for fluorescence imaging of NLs and mNPs, respectively. Biotin expression and attachment of mNPs and NLs were validated quantitatively by flow cytometry analyses with a BD FACSMelody cell sorter (BD Biosciences). A total of 10,000 events were recorded for each experiment using a blue laser source for GFP-expressing bacterial cells (fluorescein isothiocyanate: emission, 525 nm) and a yellow-green laser source for NLs and mNPs (PE-Cy5: emission, 670 nm; PE: emission, 575 nm). Bacterial biohybrids were further characterized by SEM imaging. Bacterial cells were placed on silicon wafers and allowed to sediment for 15 min to promote attachment to wafer surface. Next, cells were fixed with glutaraldehyde [2.5% (v/v), in distilled water] for 45 min at 4°C. After fixing, cells were rinsed three times with distilled water and dehydrated with increasing concentrations of ethanol solutions (25, 50, 75, 90, and 100% ethanol) and chemically dried with hexamethyldisilazane (HMDS) solutions in ethanol at increasing HMDS:ethanol (v/v) ratios (1:2, 1:1, 2:1, and 1:0). Samples were then dried overnight at room temperature. Before SEM imaging, samples were sputter-coated with 10-nm gold with a sputter coater (Leica EM ACE600, Leica Microsystems). SEM images were acquired with Zeiss Ultra 550 Gemini (Carl Zeiss Inc.) at a range of accelerating voltage of between 1 and 10 keV and an in-lens detector. For visualization purposes, SEM images were pseudocolored with Adobe Photoshop software.

### Swimming characterization and magnetic steering of bacterial biohybrids

Poly(methyl methacrylate) (PMMA) pieces and double-sided adhesive films were cut to shape with a CO_2_ laser cutter (Epilog Laser), and all components were adhered together on a cover glass to prepare microchannels into which bacterial cells were loaded. For swimming velocity and trajectory characterizations, free bacteria and bacterial biohybrids inside microchannels were tracked from video recordings acquired on an inverted optical microscope (Zeiss Axio Observer A1, Carl Zeiss) with a 40× objective lens. A minimum of 15 videos were tracked to characterize mean speed and swimming trajectories. Videos were analyzed with an in-house MATLAB code. Bacterial biohybrids were magnetically controlled with a custom-made five-coil electromagnetic setup. An inverted optical microscope (Zeiss Axio Observer A1, Carl Zeiss) was equipped with the electromagnetic coil setup to perform swimming experiments.

### Preparation of tumor spheroids

Human colorectal adenocarcinoma cells, HT-29 (American Type Culture Collection), were cultured in Dulbecco’s modified Eagle’s medium supplemented with 10% fetal bovine serum (Gibco) and 1% penicillin/streptomycin (Gibco) in a humidified, 37°C, and 5% CO_2_ environment. Cells were used at passage numbers lower than 10, and surface detachment during splitting was performed using 0.25% trypsin-EDTA (Gibco) when they reached 80% confluence. To prepare tumor spheroids, HT-29 cells were seeded into ultralow attachment 96-well spheroid microplates (Gibco) at the densities of 720 cells per well and were grown for 72 hours in a humidified, 37°C, and 5% CO_2_ environment.

### Localization of bacterial biohybrids in tumor spheroids

For magnetic guidance experiments, microchannels with three reservoirs were prepared with PMMA pieces and double-sided adhesive films on a round cover glass. The reservoirs were filled with McCoy’s 5A medium (Gibco), a single HT-29 tumor spheroid was placed in each of two reservoirs, and bacterial biohybrids were loaded in the third reservoir. The microchannel was placed on an inverted optical microscope equipped with a custom-made, 1D magnetic guidance setup with two circular permanent magnets separated by a distance of 100 mm, which generated a homogeneous magnetic field (26 mT) along one axis and directed bacterial biohybrids toward a spheroid (*16*). For magnetic gradient pulling experiments, a closed and branched microchannel (height, 100 μm; width, 200 μm) with two reservoirs was prepared with PMMA pieces, double-sided adhesive films, fluidic connections, and a cover glass as the bottom piece. PMMA pieces were cut to shape with a CO_2_ laser cutter (Epilog Laser), whereas the double-sided adhesive films were prepared using a UV laser system (LPKF ProtoLaser U3). The two reservoirs were filled with McCoy’s 5A medium (Gibco), a single HT-29 tumor spheroid was placed in each of two reservoirs, and bacterial biohybrids (8.0 × 10^7^ bacterial cells/ml; in McCoy’s 5A medium) were loaded into a syringe that was then connected to the tubing of the microfluidic channel. Bacterial biohybrids were injected to the microfluidic channel at a controlled flow velocity (15.625 μm/s) with a syringe pump (KD Scientific Inc.). A single cubic NdFeB magnet (5 mm^3^; Supermagnete) was attached to one of the reservoirs to magnetically pull bacterial biohybrids that are continuously flowing within the fluidic branches. Magnetic field strength and lines, as well as magnetic field gradient and directions, generated by the permanent magnet were simulated using COMSOL Multiphysics 5.5 (COMSOL, Burlington, MA).

### Invasion of collagen gels with bacterial biohybrids

Collagen gels were prepared with collagen stock solution [collagen type I, rat tail (5 mg/ml)] (ibidi), sterile ddH_2_O, PBS (1× and 10×) (Gibco), and 1 M NaOH to adjust pH. Final gel concentration was 2.5 mg/ml. Collagen solutions of different pH (6.5, 7.5, and 8.5) were prepared and pipetted on one side of a chambered coverslip (μ-Slide 8 Well, uncoated, ibidi) so that they only took up one-third of the total chamber volume. Gels were placed in a temperature-controlled incubator at 37°C and 5% CO_2_ for 30 min for polymerization. After polymerization, gels were thoroughly washed with fresh PBS (pH 7.4) several times to remove unreacted species and set the pH to neutral, and the rest of the chamber was filled with McCoy’s 5A cell culture medium. Next, bacterial biohybrids (10 μl, 6.0 × 10^8^ bacterial cells/ml; in McCoy’s 5A) were pipetted at the far end of the chambered coverslip. The setup was incubated overnight with the permanent magnet setup aligning the bacterial biohybrids toward the collagen gels polymerized on one corner of the chamber. Quantification of invaded bacterial cells within the collagen gels was done by using ImageJ cell counting software. A minimum of six different images were used for the quantification of the collagen gel invasion (*n* = 6).

### Collagen gel rheology

Rheological experiments were performed with Discovery Hybrid Rheometer HR-2 (TA Instruments). As the bottom geometry, an advanced Peltier plate was used, whereas for the top geometry, a 20-mm smart swap plate geometry with solvent trap was used. All experiments were conducted on oscillatory mode. Briefly, 0.45 ml of collagen solution was applied to the measuring stage set at 4°C. After descending the top geometry and placing the evaporation trap, the temperature was ramped up to 37°C at a ramp rate of 33°C/min. The sample was conditioned at 37°C for 30 min, and measurements were done at the constant frequency of 1 Hz and the constant strain of 1%.

### NIR-triggered DOX release and uptake by tumor spheroids

For measuring the possible adverse effects that could be caused by NIR irradiation, the tumor spheroids were treated with NIR light at different time intervals (5, 10, and 15 min). Then, the NIR-treated tumor spheroids were incubated in a humidified, 37°C, and 5% CO_2_ environment for 24 hours. After 24 hours of incubation, the viability of spheroids was measured using the CellTiter-Glo 3D Cell Viability Assay by using 1:1 media-to-reagent ratio. The luminescence values were measured in an opaque 96-well plate using a plate reader (BioTek’s Synergy 2, Winooski, VT, USA). The relative cell viabilities were calculated on the basis of the results obtained from the untreated group. Tumor spheroids were placed inside microwells (μ-Slide 8 Well, uncoated, ibidi). Each well contained two tumor spheroids immersed in McCoy’s 5A medium. Free bacterial cells and bacterial biohybrids (carrying DOX NLs, ICG NLs, or ICG-DOX NLs) were added to microwells (4.0 × 10^7^ bacterial cells/ml) and incubated for 10 min. Next, spheroids and bacterial biohybrids were irradiated with a NIR laser (808 nm, 0.6 W/cm^2^) for 10 min at a distance of 7 cm from the top of the microwell. Microwells were placed inside an incubator at 37°C and 5% CO_2_ for 24 hours. At the end of incubation, tumor spheroids were imaged fluorescently for DOX uptake with fluorescence and confocal microscopy. Confocal images of DOX-uptaken spheroids were taken with a Leica SP8 single-point scanning confocal microscope, and 3D reconstructions were made using LAS X software.

### Tumor spheroid viability

For tumor spheroid viability, (i) untreated tumor spheroids, (ii) tumor spheroids treated with bacterial biohybrids without NIR, and (iii) tumor spheroids treated with bacterial biohybrids with NIR for 20 min were incubated at 37°C and 5% CO_2_ in a humidified incubator overnight after respective treatments. A live/dead cell imaging solution (Invitrogen, Thermo Fisher Scientific) was used to stain 3D tumor spheroids as specified by the supplier, and they were imaged for live (excitation, 488 nm; emission, 520 nm) and dead (excitation, 528 nm; emission, 617 nm) cells under a fluorescent microscope (Nikon Eclipse Ti-E).

### Statistical analysis

All quantitative values are presented as means ± SD of the mean. Student’s *t* test was used for the statistical analysis, and statistical significance was set at 95% confidence level for all tests (*P* < 0.05).
