# Supplementary material for: Magnetically steerable bacterial microrobots moving in 3D biological matrices for stimuli-responsive cargo delivery
Source: Sci Adv. 2022 Jul 15;8(28):eabo6163. doi: 10.1126/sciadv.abo6163 (PMC9286503; doi:10.1126/sciadv.abo6163)
Supplement: Supplementary file 1 — Figs. S1 to S9 Tables S1 and S2 [file sciadv.abo6163_sm.pdf]

Science Advances
AAAS

Supplementary Materials for

Magnetically steerable bacterial microrobots moving in 3D biological matrices for stimuli-responsive cargo delivery

Mukrime Birgul Akolpoglu *et al.*

Corresponding author: Metin Sitti, sitti@is.mpg.de

Sci. Adv. **8**, eabo6163 (2022)
DOI: 10.1126/sciadv.abo6163

The PDF file includes:

Figs. S1 to S9
Tables S1 and S2
Legends for movies S1 to S7

Other Supplementary Material for this manuscript includes the following:

Movies S1 to S7

Supplementary Figures and Tables

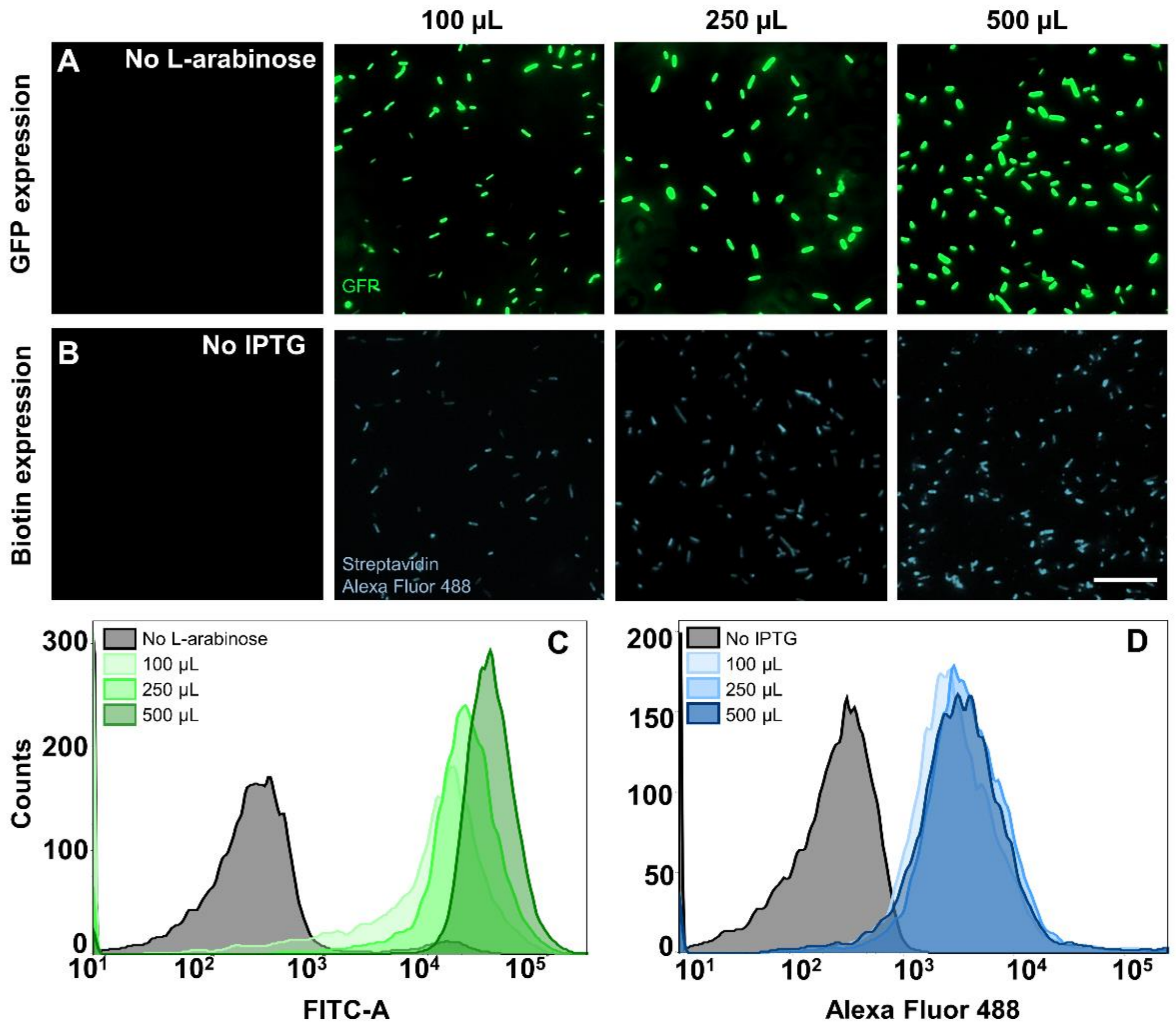

Fig. S1. Optimizations for GFP and biotin expressions. (A) Fluorescence microscopy images of bacteria induced with various amounts of *L*-arabinose (100, 250, or 500 µL) to induce GFP expression during the growth phase. (B) Fluorescence microscopy images of bacteria induced with various amounts of IPTG (100, 250, or 500 µL) to induce biotin expression. Cells were then labeled with streptavidin Alexa Fluor 488 dye for fluorescence imaging. Bacterial cells were not induced with L-arabinose in this case. Scale bar, 25 µm. (C) Flow cytometry analysis for bacterial cells induced with or without various amounts of *L*-arabinose. (D) Flow cytometry analysis for bacterial cells induced with or without various amounts of IPTG.

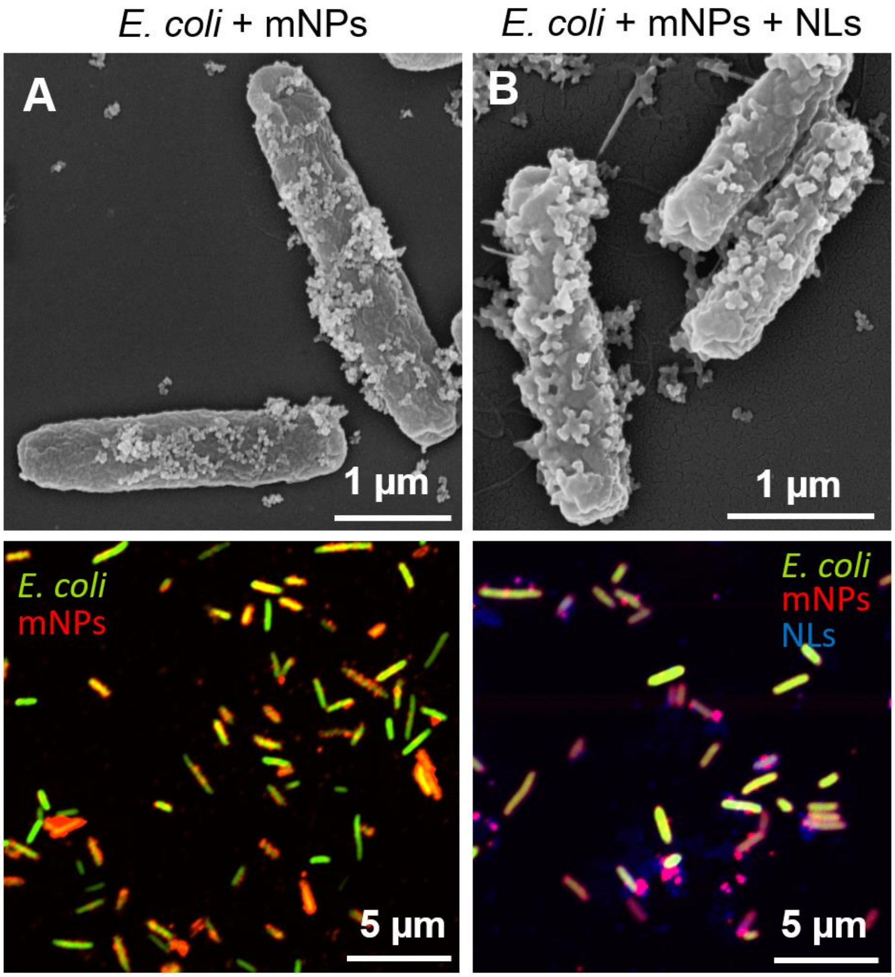

Fig. S2. Imaging characterization of bacterial biohybrids. (A) SEM and fluorescence microscopy images of bacterial biohybrids conjugated with fluorescent mNPs. (B) SEM and fluorescence microscopy images of bacterial biohybrids conjugated with fluorescent mNPs and NLs. Green: bacteria, red: mNPs, and blue: NLs.

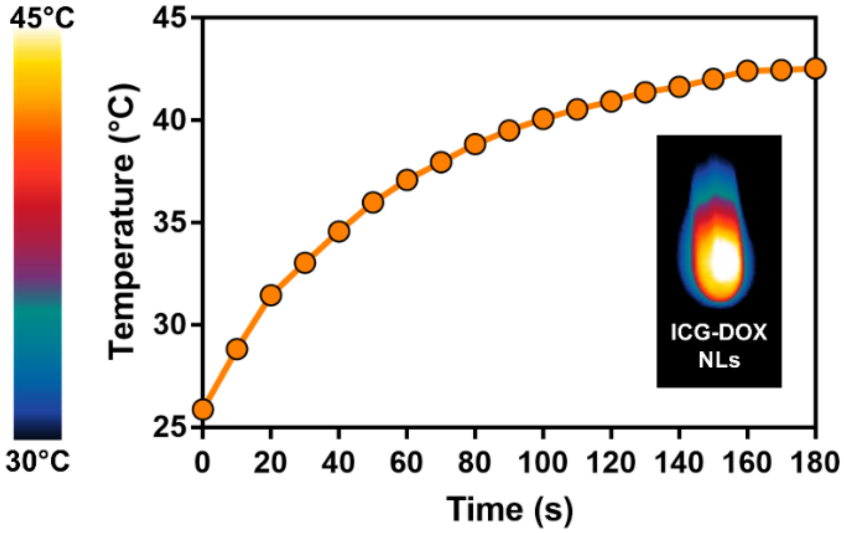

Fig. S3. Infrared thermal image and temperature profile of ICG-DOX NLs under NIR irradiation. ICG-DOX NLs were illuminated with NIR light (~0.6 W cm^{-2}, 180 s) and the change in temperature was plotted against time. Inset represents the infrared thermal image of the heated liposome solution.

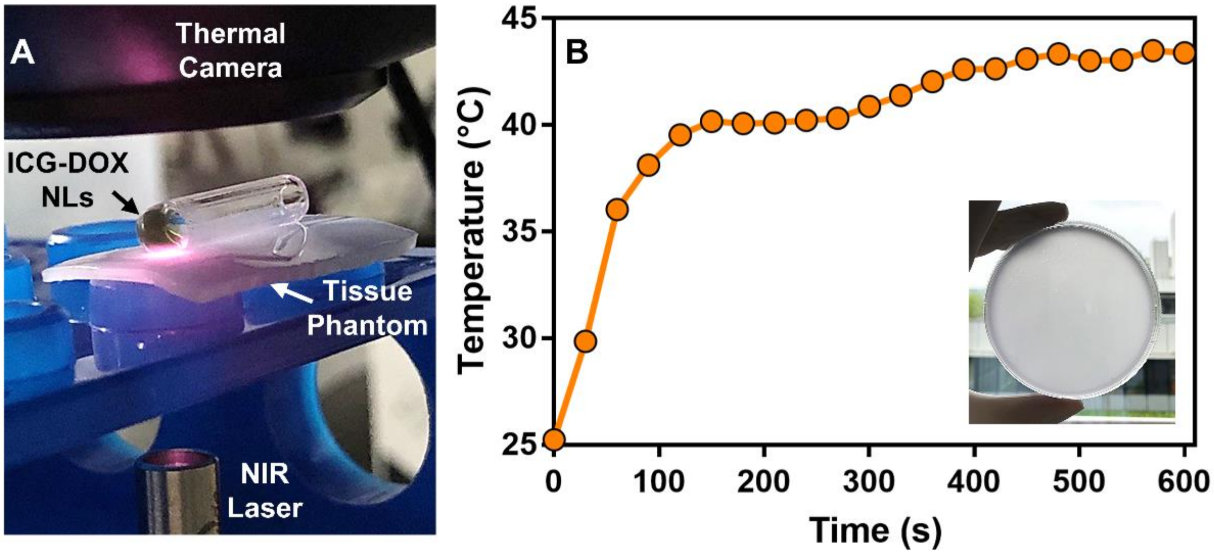

Fig. S4. Photothermal heating experiments with a tissue phantom. (A) Experimental setup with a thermal camera, a glass tube with ICG-DOX NLs, a 1 mm-thick slice of a tissue phantom, and an NIR laser probe. (B) Temperature profile of ICG-DOX NLs irradiated through tissue phantom with NIR (~0.6 W cm^{-2}, 180 s). Inset shows the tissue phantom.

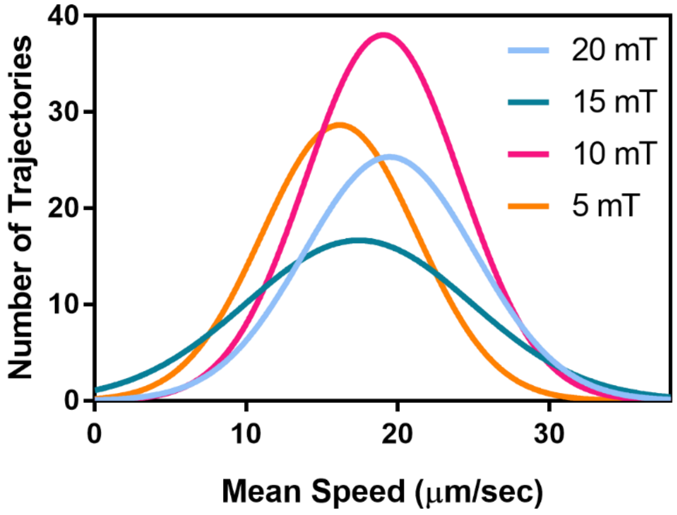

Fig. S5. 2D swimming velocity analyses of bacterial biohybrids carrying mNPs with applied external magnetic fields (5, 10, 15, and 20 mT). The measured velocities were recorded at 16.3 ± 4.1 µm/s, 18.5 ± 4.7 µm/s, and 16.4 ± 1.7 and 17.7 ± 2 µm/s for the applied magnetic field of 5, 10, 15 and 20 mT, respectively.

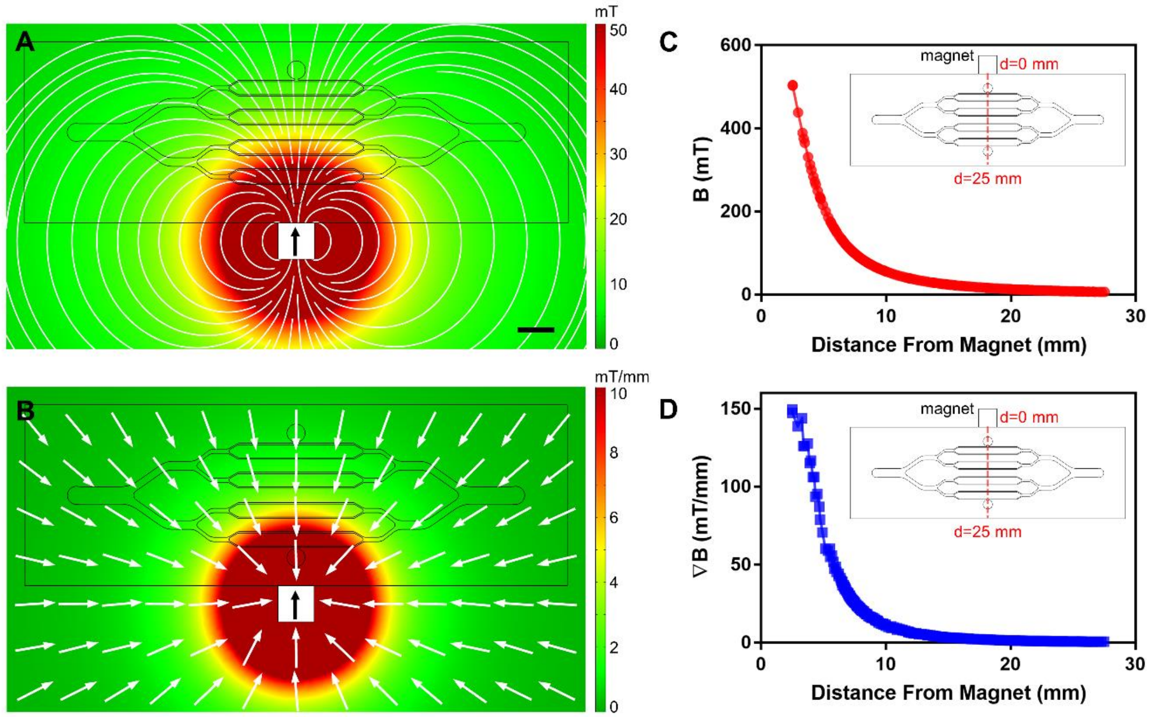

Fig. S6. Simulation of magnetic field strength, gradient, and directions generated by a cubic permanent magnet placed next to a branched microfluidic channel. (A) Magnetic field strength and field lines (white lines) and (B) magnetic field gradient and directions (white arrows) over the branched microfluidic channel setup. Color bars indicate magnetic field strength and magnetic gradient, respectively. Black arrows within the magnets indicate magnetization direction of the permanent magnets. Scale bar, 5 mm. (C, D) Magnetic field, B, and magnetic field gradient, ∇B, plotted against distance from the permanent magnet through the center of the microfluidic channel, displaying negligible field and gradient strength in the reservoir at the other end of the channel setup.

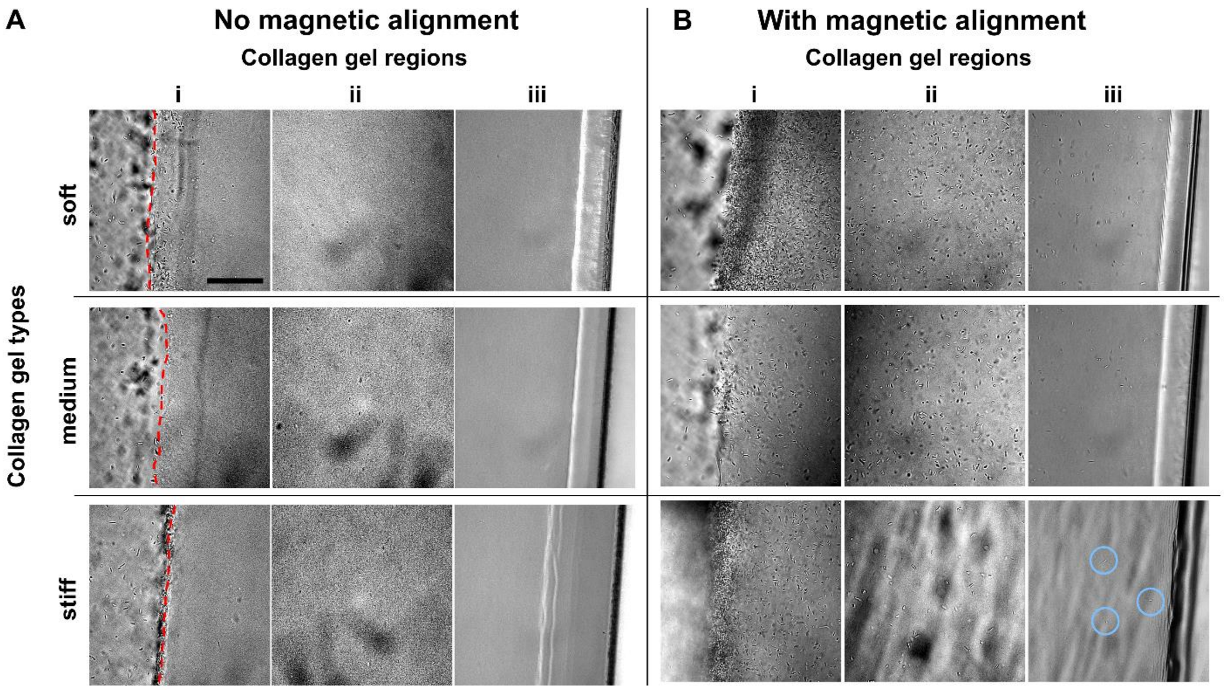

Fig. S7. Brightfield microscopy images of collagen gel regions after overnight incubation with bacterial biohybrids, (A) with and (B) without magnetic field. Gels were divided into three regions: i, ii, and iii. Region i represents the initial region right after liquid-gel interphase (shown with red dashed line): ii represents the middle region, and iii represents the deepest end of the collagen gel. Blue circles represents the bacterial biohybrids detected at region iii on the rigid gel after with magnetic alignment. Scale bar: 50 µm.

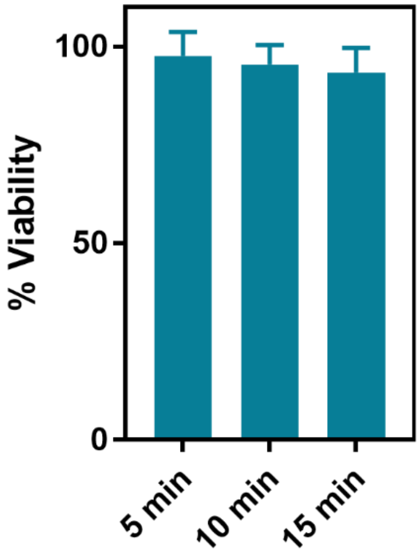

Fig. S8. Percent viabilities of HT-29 tumor spheroids after NIR-irradiation for predetermined durations (5, 10, and 15 min). A luminescent cell viability assay (Cell Titer Glo) was performed 24 h after NIR-light irradiation.

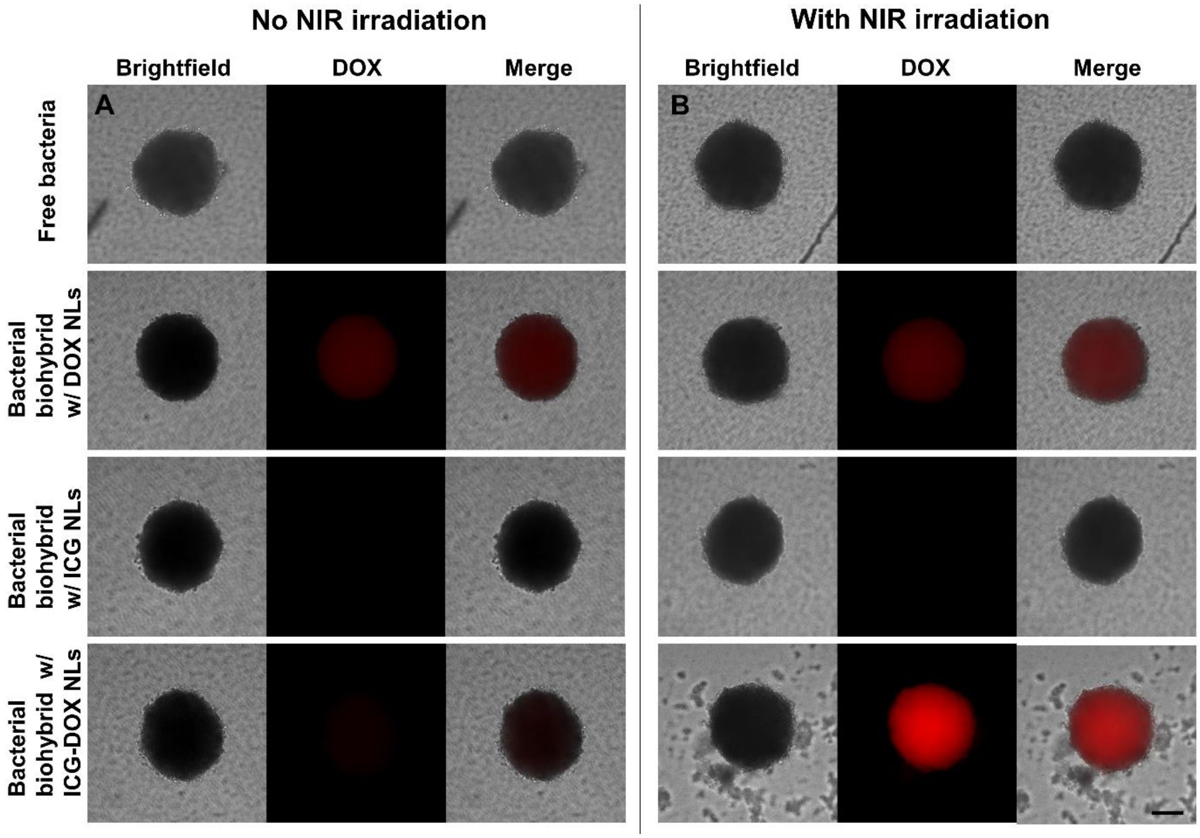

Fig. S9. Microscopy images of all spheroids co-incubated with free bacteria or bacterial biohybrids carrying different liposomal cargoes, (A) without or (B) with NIR irradiation. Images were captured 24 h after NIR irradiation. Red fluorescence indicates the presence of DOX. Scale bar: 100 µm.

Table S1. DOX loading optimization by changing DOX:lipid mass ratio and lipid concentrations

**Lipid concentration**	**DOX:phospholipid mass ratio**	**Encapsulated DOX concentration (μg/mL ± SD)**	**Encapsulation efficiency (EE %)**
1.56 mg/ml	1:5	38 (2.1)	60.7 %
1.56 mg/ml	1:10	86.1 (3.4)	82.4 %
3.12 mg/ml	1:5	30.2 (4.5)	48.2%
3.12 mg/ml	1:10	85.6 (1.8)	81.9%

Table S2. Average hydrodynamic diameters and zeta potentials of different liposome formulations

	**Average hydrodynamic diameter (nm ± SD)**	**Zeta potential (mV)**
**Blank liposomes**	190 (20)	+8.3
**DOX liposomes**	275 (14)	+8.4
**ICG liposomes**	233 (23)	+7.8
**DOX+ICG liposomes**	205 (13)	+7.6

Supplementary Movies

Movie S1. Swimming of bacterial biohybrids carrying magnetic nanoparticles (mNPs) and nanoliposomes (NLs)

Movie S2. Swimming of bacterial biohybrids under continuously applied magnetic field (10 mT)

Movie S3. Bacterial biohybrids swimming towards tumor spheroid under constant magnetic guidance

Movie S4. Bacterial biohybrids swimming towards HT-29 tumor spheroid under magnetic field gradients in flow conditions

Movie S5. Bacterial biohybrids navigating inside a medium collagen gel under constant magnetic alignment

Movie S6. Bacterial cells swarming around a HT-29 tumor spheroid

Movie S7. mCherry expressing bacterial biohybrids swimming along applied magnetic field
